# Cranial Anatomy of the Earliest Marsupials and the Origin of Opossums

**DOI:** 10.1371/journal.pone.0008278

**Published:** 2009-12-16

**Authors:** Inés Horovitz, Thomas Martin, Jonathan Bloch, Sandrine Ladevèze, Cornelia Kurz, Marcelo R. Sánchez-Villagra

**Affiliations:** 1 Department of Ecology and Evolutionary Biology, University of California Los Angeles, Los Angeles, California, United States of America; 2 Steinmann-Institut für Geologie, Mineralogie und Paläontologie, Universität Bonn, Bonn, Germany; 3 Florida Museum of Natural History, University of Florida, Gainesville, Florida, United States of America; 4 Palaeontologisches Institut und Museum, Zürich, Switzerland; 5 Naturkundemuseum im Ottoneum Kassel, Kassel, Germany; American Museum of Natural History, United States of America

## Abstract

**Background:**

The early evolution of living marsupials is poorly understood in part because the early offshoots of this group are known almost exclusively from jaws and teeth. Filling this gap is essential for a better understanding of the phylogenetic relationships among living marsupials, the biogeographic pathways that led to their current distribution as well as the successive evolutionary steps that led to their current diversity, habits and various specializations that distinguish them from placental mammals.

**Methodology/Principal Findings:**

Here we report the first skull of a 55 million year old peradectid marsupial from the early Eocene of North America and exceptionally preserved skeletons of an Oligocene herpetotheriid, both representing critical groups to understand early marsupial evolution. A comprehensive phylogenetic cladistic analysis of Marsupialia including the new findings and close relatives of marsupials show that peradectids are the sister group of living opossums and herpetotheriids are the sister group of all living marsupials.

**Conclusions/Significance:**

The results imply that North America played an important role in early Cenozoic marsupial evolutionary history and may have even been the center of origin of living marsupials and opossums. New data from the herpetotheriid postcranium support the view that the ancestral morphotype of Marsupialia was more terrestrial than opossums are. The resolution of the phylogenetic position of peradectids reveals an older calibration point for molecular estimates of divergence times among living marsupials than those currently used.

## Introduction

Extant marsupials are limited mostly to Australia and South America whereas the few Central and North American representatives are relatively recent immigrants from South America through the Panamanian Isthmus circa 3 million years ago. Fossil relatives of marsupials are common in Cretaceous through Miocene localities in both North America and Eurasia [1], [2]. Metatheria includes the common ancestor of all extant marsupials plus all extinct mammals that are more closely related to living marsupials than to extant placentals [3]. Recent discoveries [3], [4] have enhanced our understanding of the origin and early evolution of Metatheria. In contrast, the early evolution of living marsupials and their closest relatives remains poorly known. The origin of opossums, the Didelphidae, is of particular significance because this group resulted from the first cladogenetic event of Marsupialia [5]-[7]. Herpetotheriidae were believed to be close relatives of didelphid opossums until recently when this group was shown to be a close relative of Marsupialia instead [8], [9].

Peradectidae is another key fossil metatherian group for the question of marsupial origins. It has been alternatively considered to be a member of Didelphidae or of a paraphyletic ‘Didelphimorphia’ [10] or their close relative [11]–[13], an unresolved basal branch of Marsupialia [14], a close relative of Microbiotheria [15], or a stem metatherian offshoot [16]. Peradectidae are predominantly from the Northern Hemisphere, with a fossil record starting at least in the early Paleocene with *Peradectes*. This genus continues into the Eocene of North America [12] and it also has Paleocene South American [1], [17] and Eocene European representatives [18]–[20]. The fossil record of peradectids extends until the early Miocene of North America with *Nanodelphis* [21]. In the past some Cretaceous forms were included in the Peradectidae, such as the important stem marsupial *Alphadon* [22]. However, this and other dentally superficially similar Mesozoic taxa are excluded from Peradectidae following current hypotheses based on dental features [12], [13], [21]. For example, Johanson [13] had hypothesized shared-derived dental features of peradectids (as treated here) and living opossums (Didelphidae) to the exclusion of Cretaceous forms. Ideas involving the timing of early marsupial evolution and the relationships of basal groups of cosmopolitan distribution need testing with comprehensive analyses of better fossils. This is now possible based on new fossils of this group, such as *Mimoperadectes labrus,* from the earliest Eocene of the Clark Fork Basin, Wyoming [11]. Here we describe a fairly complete skull of a new species of *Mimoperadectes* and provide new information on herpetotheriids based on exceptional skeletons. All this leads to the first scientific restorations of these animals and an informed consideration of their paleobiology in a tested phylogenetic framework.

## Results and Discussion

Mammalia Linnaeus 1758

Metatheria Huxley 1880

Marsupialia Illiger 1811

Peradectidae Crochet 1979

*Mimoperadectes* Bown and Rose 1979

***Mimoperadectes houdei*** sp. nov.

urn:lsid:zoobank.org:act:CA1AEA7A-C974-463E-B8E9-A49911AB5082

### Etymology

Named for Dr. Peter Houde from New Mexico State University who, in the process of studying limestone avifauna from the Clark Fork Basin, has discovered and prepared many spectacular early Eocene mammal specimens including the type specimen of *Mimoperadectes houdei*.

### Holotype

**Figure 1:**
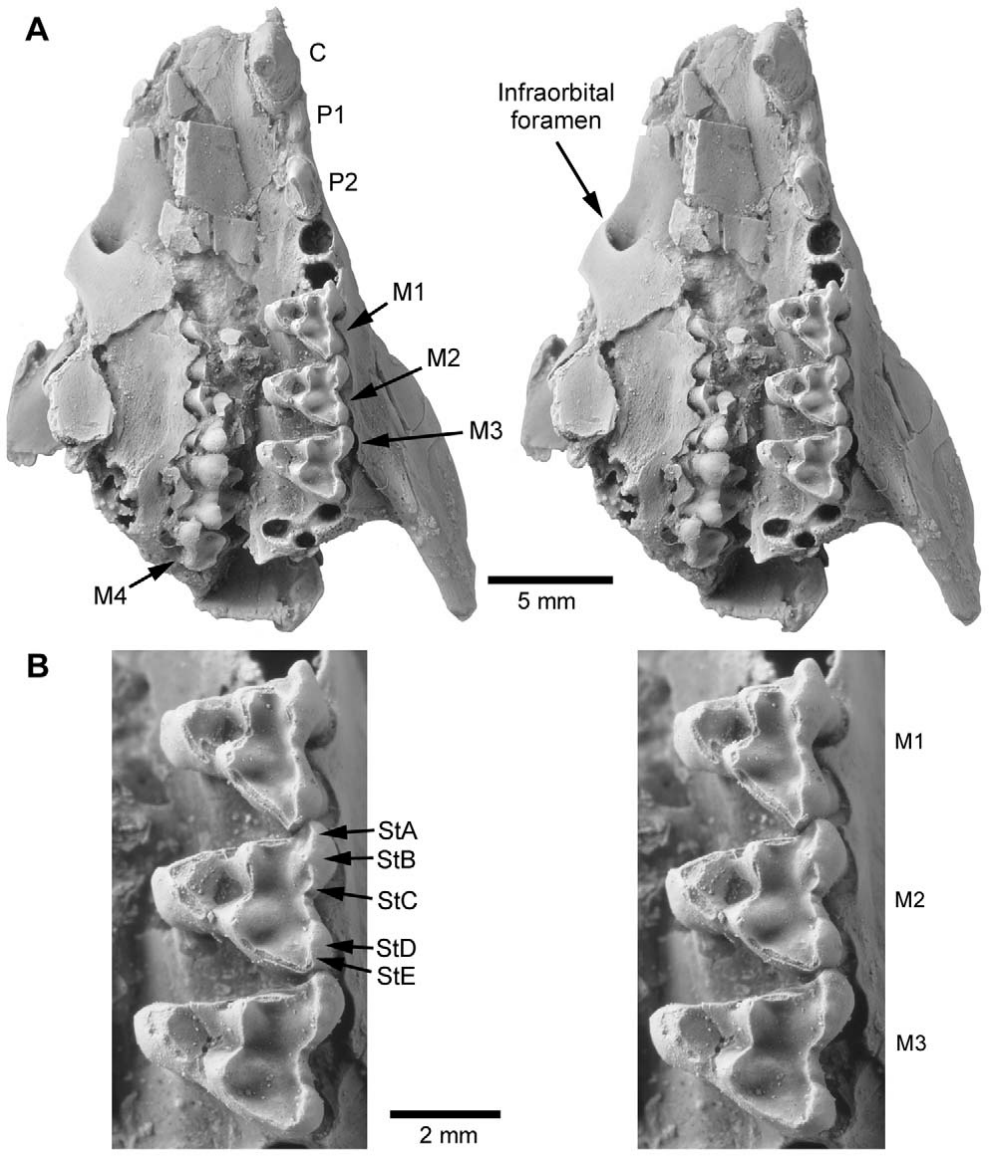
Skull of *Mimoperadectes houdei* USNM 482355. Stereo photographs of (A) anterior fragment in ventral view and (B) M1-3 in occlusal view.

**Figure 2:**
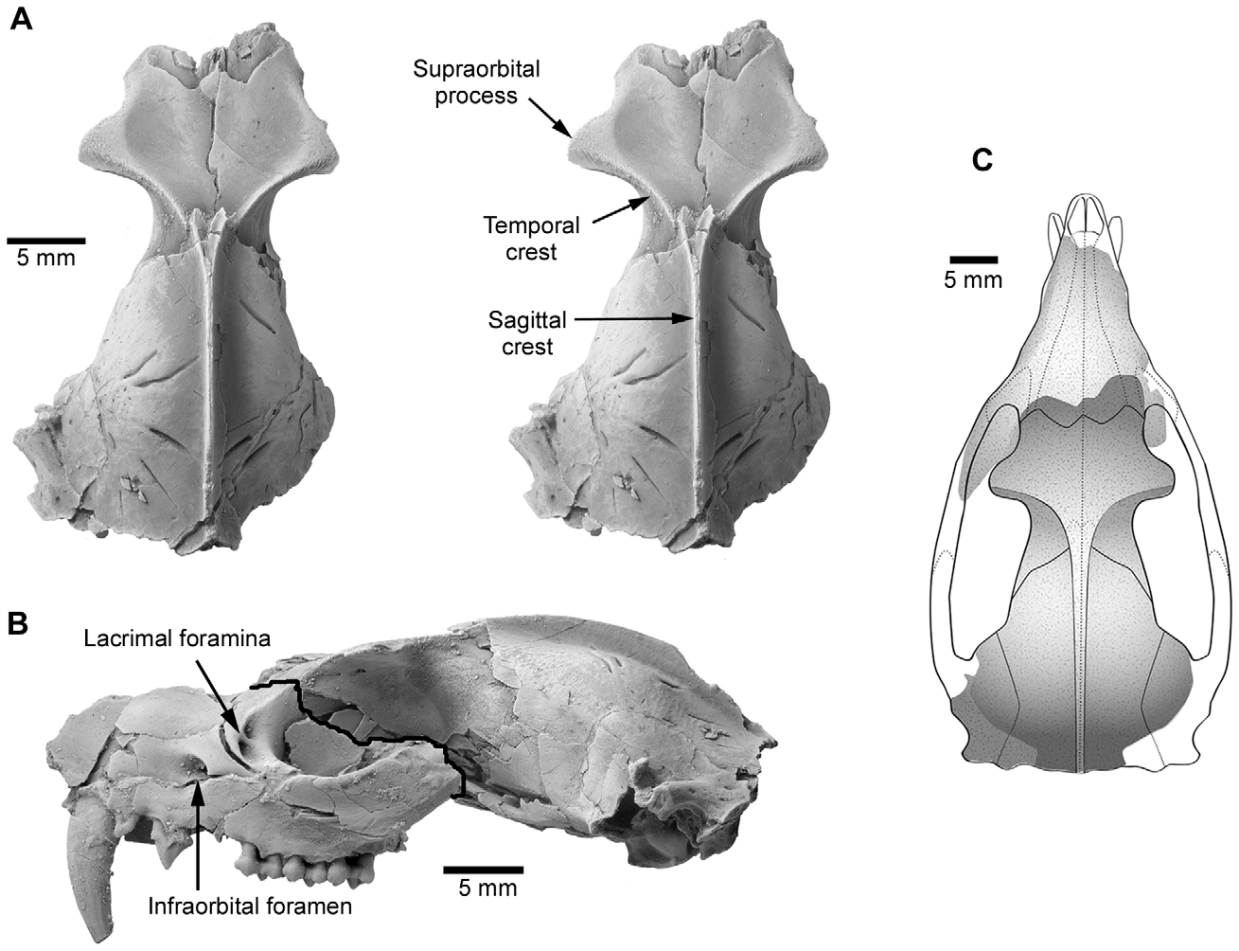
Skull of *Mimoperadectes houdei* USNM 482355. (A) Posterior fragment in dorsal view. (B) Composite of two left-view photographs of anterior and posterior fragments separated by a thick line. (C) Schematic reconstruction joining both fragments in dorsal view. Abbreviations: C, canine; P, premolar; M, molar; St, stylar cusp.

**Figure 3:**
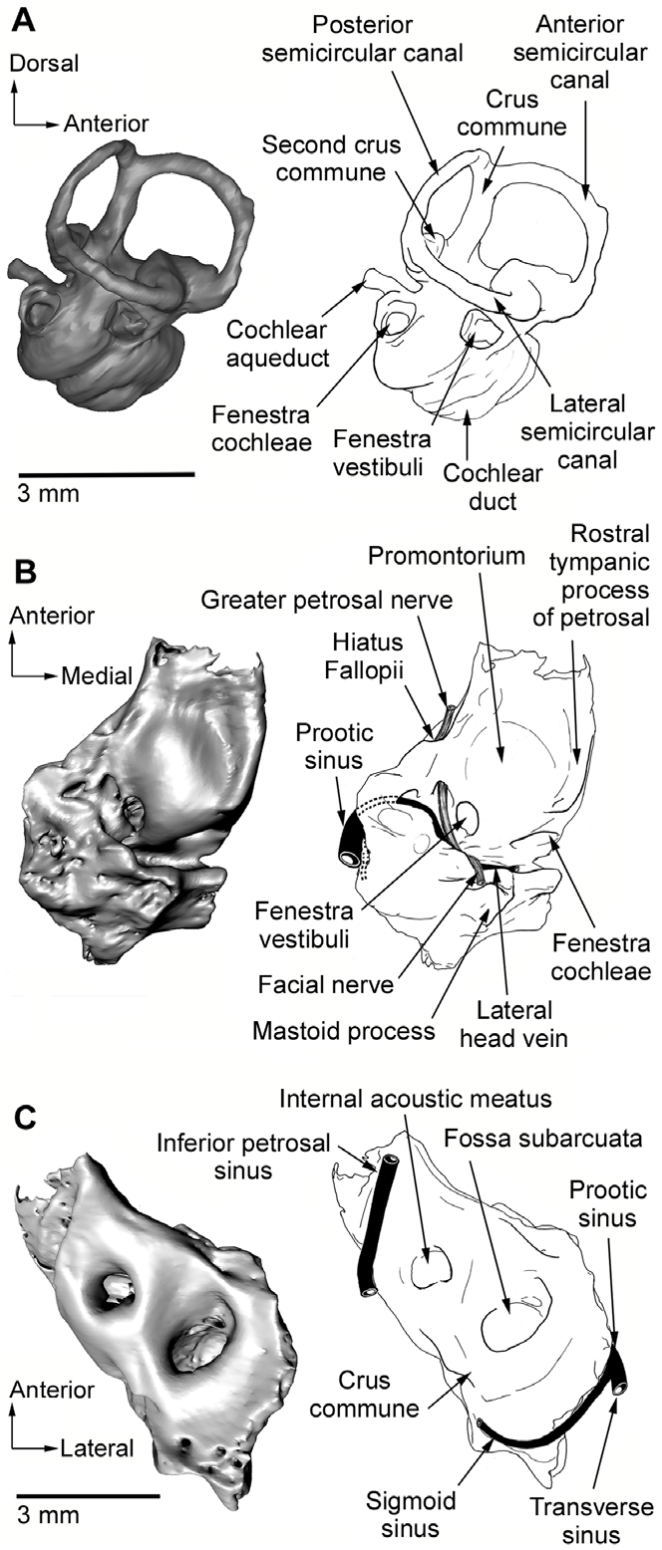
Computed tomography (CT) reconstruction and keys. (A) Right bony labyrinth in lateral view and (B–C) right petrosal in ventral and dorsal views respectively, with reconstruction of arteries, veins, and nerves.

United States National Museum of Natural History (USNM) 482355, a broken cranium separated into one anterior and one posterior piece (Figure 1, 2, S1, S2, S3, S4). The anterior portion is a partial rostrum that preserves left C, P1-2, M1-3 and right M1-4. The posterior portion consists of the braincase and parts of the basicranium with a complete middle and inner ear (Figure 2, 3, S5).

### Type Locality

UM locality SC-133 is located in the northern half of the NE1/4, Section 1, T55N, R102W, Park County, Clark Fork Basin, Wyoming [23].

### Horizon and Age

Type and only known specimen prepared from a freshwater limestone nodule [24] from the lower part of the Willwood Formation; Early Wasatchian (uppermost *Arfia shoshoniensis* interval zone, early Eocene, between 54.7–54.4 Mya [25]).

### Diagnosis

Differs from the type and only other species of *Mimoperadectes*, *M. labrus*, in having: (1) M1 longer than M2, with a larger surface area than the M2 whereas the reverse is true in *M. labrus*; the surface ratio between M1 and M2 is about 20% larger in *M. houdei* than in *M. labrus*; (2) M1-3 midline anteroposterior constriction, with the lingual half of the crown relatively narrower, especially noticeable on M3; (3) protocone lobe lingually expanded in M1-3; (4) metacone reduced in M4.

### Description

*Mimoperadectes* has prominent supraorbital processes, substantially more developed than in *Didelphis* but similar to *Caluromys*. The postorbital constriction is relatively wider in *Mimoperadectes* than in *Didelphis* although probably not as wide as in *Mayulestes.* A sagittal crest starts slightly posterior to the postorbital constriction at the junction of well-defined temporal crests. Sutures of the braincase are obliterated in the holotype. The anterior root of the zygomatic process extends from the middle of M1 to the posterior end of M4. (Figure 1A). Despite some damage, two lacrimal foramina can be detected. The infraorbital foramen is located above the posterior end of P2 and anterior border of the alveolus for P3. A poorly-developed tympanic wing of the alisphenoid is present as in *Didelphis* and *Monodelphis*. The foramen ovale is surrounded by the alisphenoid and the petrosal. The foramen for the transverse canal is in a position anterior to the carotid foramen. The petrosal of *Mimoperadectes* displays the following characteritics: the mastoid process is small and slanted; the mastoid exposure on the occiput is narrow; the rostral tympanic process forms a distinct crest; the prootic canal is retained, as well as the posttemporal sulcus (for the diploetic vessels) on the squamosal surface of the petrosal (see Text S1, S2 for more information). The number of cochlear turns is close to that of didelphids (2.1 in *Mimoperadectes* and 2.4–2.5 didelphids). *Mimoperadectes* exhibits a ventral hiatus Fallopii, similar to that of certain australidelphians (e.g. peramelians, phalangerids, macropodids).

The dentition is only slightly worn. Although M1-3 are preserved on both sides, the right series is displaced and the occlusal surfaces are not visible. M4 is only preserved on the right side and its occlusal surface is only visible at a tight angle (Figure S5). All four molars are wider than longer, and although M4 is the shortest and narrowest of them all, proportions among the other three vary greatly (Table S1, Text S3). The order of size for M1-3 from shortest to longest is M2<M3<M1, whereas the order from narrowest to widest is M1<M2<M3. In contrast, the widths of M1-3 are almost even in *Mimoperadectes labrus*, whereas the length of the molars increases sequentially from M1 to M3.

The M1 paracone is slightly smaller than the metacone on M2-3 and the stylar cusp B is the largest stylar cusp in both *M. houdei* and *M. labrus*. The presence of a paraconule and metaconule cannot be verified in USNM 482355 because of wear but if present, they were most likely small, as in *M. labrus*. The metastylar area of M4 in *M. houdei* is narrow whereas it is practically absent in *M. labrus*. The two species are much larger in overall size than *Peradectes*.

### Phylogenetic Analysis and Implications of Results

**Figure 4:**
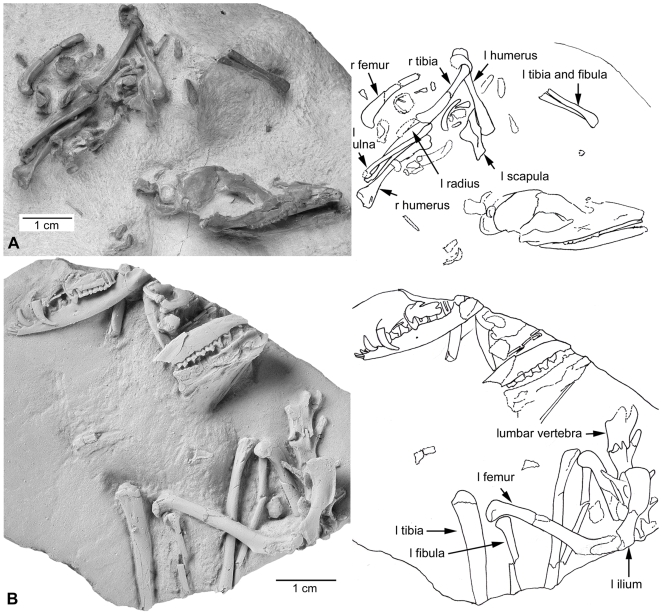
Photographs and keys of *Herpetotherium cf. fugax*. (A) SMF 2000/168. (B) SMF 2000/169. Abbreviations: l, left; r, right; SMF, Senckenberg Museum Frankfurt.

**Figure 5:**
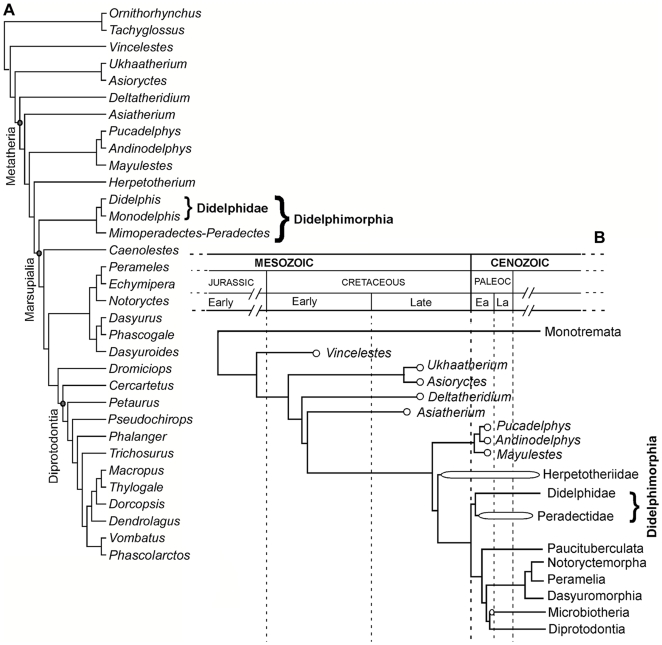
Phylogenetic relationships and ages. (A) Most parsimonious cladogram resulting from analysis of morphological data matrix, of length = 1013, CI = 0.43, RI = 0.65, RC = 0.28 (see SI for data matrix and branch support values). (B) Summary diagram of ‘A’ showing minimum ages of cladogenetic events (after Luo [43], [44] and evidence presented here). Empty balloons represent fossil species. Abbreviations: Paleoc, Paleocene; Ea, Early; La, Late.

We conducted a phylogenetic analysis incorporating morphological data from *Mimoperadectes* and *Peradectes* (Text S2, Table S2). We also included two new specimens of *Herpetotherium* with almost complete forelimbs and several posterior vertebrae, the anatomy of which was barely known before for the clade (Figure 4). The allocation of these specimens to *Herpetotherium* is based on dental features such as presence of procumbent lower incisors and the large size of the stylar cusp C in M3 [26]. Furthermore, the postcranial elements of these specimens are similar in size and morphology with those already described for *Herpetotherium* cf. *fugax* [9]. The analysis yielded a single most-parsimonious cladogram (Figure 5, S6, Table S3). The peradectids are the sister group of didelphids. *Herpetotherium* is the sister group of crown Marsupialia, confirming earlier results [8], [9].

Some classic characters used in therian systematics relate to the bony composition of a tympanic bulla (or floor for the middle ear). Among metatherians, the presence of an alisphenoid wing in the tympanic bulla is the typical condition for Marsupialia and is absent from *Mayulestes*, *Andinodelphys*, and *Pucadelphys* and outgroups used in this analysis (as well as from most eutherians). New data from the skulls of both *Mimoperadectes* and *Herpetotherium* included in our phylogenetic analysis indicate the appearance of this structure in the common ancestor of *Herpetotherium* and Marsupialia, where it was probably poorly or moderately developed.

Other derived characters supporting a clade composed of *Herpetotherium*, peradectids and other marsupials include: (1) a reduced to absent metaconule on the upper molars, (2) a cavum epiptericum that is primarily or exclusively floored by the alisphenoid, (3) a rostral tympanic process of the petrosal forming a distinct crest, and (4) a tympanic sinus that is almost entirely formed by the alisphenoid.

The close relationship of peradectids and Didelphidae, a clade we will call Didelphimorphia, is supported by the shifting of the tympanic aperture of the hiatus Fallopii from a dorsal position to an intermediate, more ventral position in the ancestral didelphimorph (and further shifted ventrally in peradectids). In addition, the diploetic vessels are present as a posttemporal sulcus and a notch, derived from being absent. Peradectids and didelphids are primitive in retaining a small and slanted mastoid process that practically disappears in other marsupials.

A reconstruction of the osseous inner ear shows that *Mimoperadectes* has fewer cochlear turns (2.1) than the two didelphids with osseus inner ear known to date (2.4–2.5) [27], [28], but more than *Herpetotherium* (1.6) [8], [9].The bony labyrinth exhibits a peculiar feature (also found in some other metatherians [8], [9], [28]) which is the presence of a second crus commune, formed by the the posterior arm of the lateral semicircular canal and the inferior arm of the posterior semicircular canal.

In light of our results, the fossil record of living marsupials starts with Puercan *Peradectes* (at least about 65.18 million years old, Early Paleocene of North America) [12], [29], [30], which implies that the basal splitting of Marsupialia into Didelphimorphia and all other marsupials had already happened by that time (Figure 5). Evolutionary splitting events within Marsupialia have been estimated using hypothetical models of DNA evolution calibrated with various fossils, all of which are younger than *Peradectes* [6], [7], [31]. The Puercan *Peradectes* offers a new factual reference point for the timescale of marsupial and therian evolution.

The minimum age for the splitting event that gave rise to crown group Marsupialia and its sister group Herpetotheriidae would be equal to the age of the oldest member of either group, namely the herpetotheriid *Nortedelphys magnus* from the late Campanian (Late Cretaceous) of North America, roughly 75 million years old [16].

Relationships among marsupial orders resulting from our analysis show remarkable congruence with recent results based on nuclear DNA sequences except for the branching order between *Dromiciops* and clade ‘A’ (*Notoryctes*+Dasyuromorphia + Peramelia) and for the position of Peramelia which is variable within clade ‘A’ [7], [32], [33]. The position of *Dromiciops* nested within the Australasian radiation in our results is in agreement with recent analyses incorporating critical early australidelphians [34] and with a simultaneous analysis of DNA sequences and morphological data [5].

There is strong evidence that metatherians originated in either Asia or North America [4], [35] and that some basal metatherians closely related to crown marsupials inhabited South America [36]. Several biogeographic scenarios are compatible with our phylogenetic results and it is clear that North America was a place of diversification and possibly even origin of marsupials and didelphimorphians.

**Figure 6:**
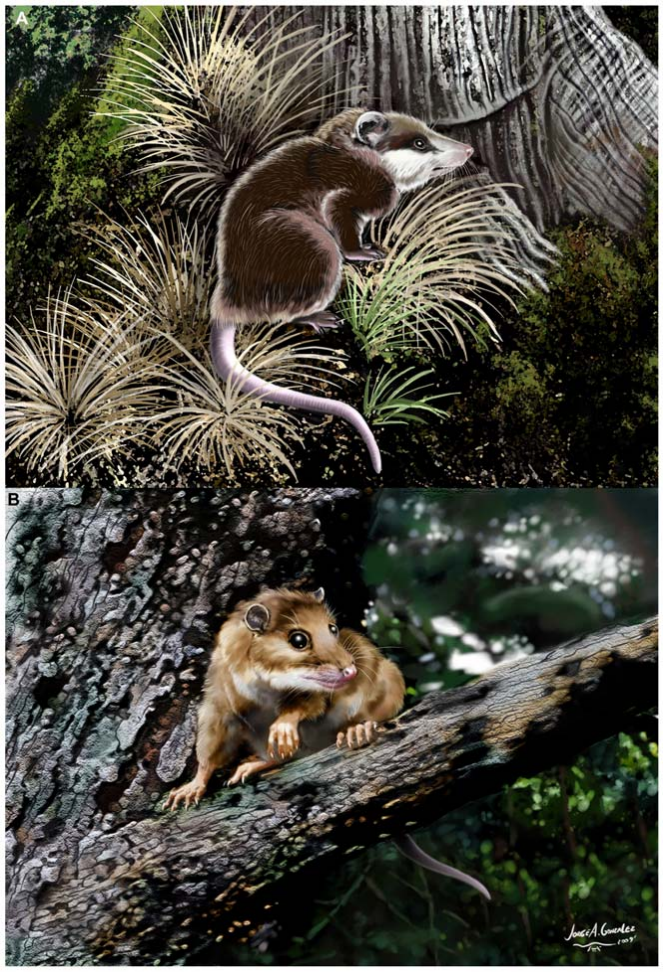
Restorations of (A) *Herpetotherium* and (B) *Mimoperadectes*, by Jorge González (La Plata, Argentina).

The new specimens of *Herpetotherium* shed light into the early evolutionary steps in the postcranium of marsupials. The forelimbs are remarkably gracile in *Herpetotherium* and indicate stronger terrestrial habits than those in didelphids, European peradectids, and the Paleocene stem metatherians *Mayulestes*, *Pucadelphys* and *Andinodelphys*, which themselves have been inferred to have been more terrestrial and displayed greater agility than didelphids [37]–[40]. The processes of the lumbar vertebrae of *Herpetotherium* are long, anteroposteriorly slender and with an anterior inclination, similar to those in *Mayulestes* and *Pucadelphys* and in contrast with those of many didelphids where the spinous processes are low and anteroposteriorly wide. This morphology suggests that *Herpetotherium* had a lower back that allowed more flexion and extension also typical of terrestrial habits, which is consistent with previous findings based on the hindlimb of *Herpetotherium* [8], [9] and with locomotory inferences concerning another herpetotheriid, *Amphiperatherium* from theMiddle Eocene of Messel, Germany [18]–[20]. On the other hand, the postcranium of peradectids from the same European locality represents a scansorial/arboreal ecotype [18]–[20], closer in functionality to living didelphids. Our results, coupled with the fact that caenolestids are mostly terrestrial animals [41] suggest that the ancestral morphotype of Marsupialia, as well as that of the *Herpetotherium*+Marsupialia clade, were more terrestrial than didelphids and than the ancestor of didelphimorphia. However if we consider the contrary hypothesis where *Dromiciops* can be the sister group of Australidelphia [7], the condition for the ancestral marsupial would be ambiguous and a tendency for arboreality would be as parsimonious as a more terrestrial lifestyle. *Dromiciops* is arboreal and although it has different pedal specializations from didelphids [41], both clades may have inherited arboreal habits from a common ancestor and subsequently each acquired its own arboreal specializations. The only available comprehensive cladistic treatments of marsupials and other metatherians that incorporate any herpetotheriids including this one, suggest that among metatherians with preserved postcranial skeletons, herpetotheriids are the closest relatives of crown Marsupialia [8], [9]. Previous studies of postcranial adaptations in basal stem-metatherians suggested aboreal habits for at least some species [4] and developement of terrestrial habits in other later stem metatherians [37]–[40]. All this information means undoubtly that in the long history of metatherian evolution from the ca. 125 Ma *Sinodelphys* to the origin of the crown group around 65 Ma [6], different kinds of locomotory habits evolved, indicating that those habits observed in basal living didelphids may not have been a direct inheritance from the ancestral marsupials. Restorations of *Herpetotherium* and *Mimoperadectes* (Figure 6) serve to graphically summarize the functional implications of our work and with that emphasize some of the diversity that existed in the dawn of marsupial and opossum evolution in North America.

## Materials and Methods

The phylogenetic analysis included 28 Mesozoic, Tertiary and Recent metatherians and five outgroups scored for 260 characters of the skull, dentition, postcranium and soft-tissues wherever possible. *Mimoperadectes houdei* is by far the bestknown peradectid in terms of anatomy, therefore it was chosen as the main representative for Peradectidae in the phylogenetic analysis. Some elements of its dentition are unknown, however, so codings for this species were complemented with codings from the dentition of *Mimoperadectes labrus* and *Peradectes* (see SI for more details). We were also able to include three postcranial characters from cf. *Peradectes* from the Middle Eocene of Messel, Germany.

The data matrix was submitted to a heuristic search using maximum parsimony with PAUP* 4.0b10 for Unix [42]. We conducted 1,000 replicates of a heuristic search with stepwise random addition sequence of taxa and tree bisection-reconnection. See Figure S6 and explanation in Text S2 for support measures.

The electronic version of this document does not represent a published work according to the International Code of Zoological Nomenclature (ICZN), and hence the nomenclatural acts contained herein are not available under that Code from the electronic edition. A separate edition of this document was produced by a method that assures numerous identical and durable copies, and those copies were simultaneously obtainable (from the publication date listed on page 1 of this article) for the purpose of providing a public and permanent scientific record, in accordance with Article 8.1 of the Code. The separate print-only edition is available on request from PLoS by sending a request to PLoS ONE, 185 Berry Street, Suite 3100, San Francisco, CA 94107, USA along with a check for $10 (to cover printing and postage) payable to “Public Library of Science”.

The online version of the article is archived and available from the following digital repositories: PubMedCentral (www.pubmedcentral.nih.gov/), and LOCKSS (http://www.lockss.org/lockss/). In addition, this published work and the nomenclatural acts it contains have been registered in ZooBank (http://www.zoobank.org/), the proposed online registration system for the ICZN. The ZooBank LSIDs (Life Science Identifiers) can be resolved and the associated information viewed through any standard web browser by appending the LSID to the prefix “http://zoobank.org/”.

## Supporting Information

Text S1 Anatomical description of the petrosal of *Mimoperadectes houdei* USNM 482355
(0.07 MB DOC)

Text S2 List of characters and phylogenetic analysis
(0.22 MB DOC)

Text S3 Cranial and dental measurements and notes about Table S1
(0.03 MB DOC)

Table S1 Dental measurements. W = width, L = length, ratio = W/L, P = upper premolar, M = upper molar, left teeth except for M4. Measurements in milimeters taken with a Zeiss Discovery V12 Stereomicroscope with an Axiocam digital Camera and Axiovision software. See Text S3 for source of *Mimoperadectes labrus* measurements
(0.03 MB DOC)

Table S2 Data matrix. Polymorphic entries, a = (01), b = (02), c = (12), d = (012). Inapplicable entries are represented with “- ” and unknown as “?”. *Mimo/peradectes* stands for *Mimoperadectes-Peradectes*
(0.29 MB DOC)

Table S3 List of unambiguous synapomorphies
(0.25 MB DOC)

Figure S1 Photographs of anterior portion of the skull of *Mimoperadectes houdei* in (A) dorsal (stereo photographs), (B) right and (C) left lateral views. Abbreviations: C, canine; P, premolar: M, molar
(5.00 MB TIF)

Figure S2 Anterior portion of skull and molars of *Mimoperadectes houdei*. (A) Drawing of anterior portion of skull in ventral view, at a slight angle from photographs in Figure 1A, (B) drawing of left molars M1-3 of in occlusal view, at a slight angle from photographs in Figure 1B, (C) photograph of right M4 in posterobuccal view. (Displacement of this molar during preservation prevents a straight buccal view and use of a scalebar). Abbreviation: St, stylar cusp
(1.39 MB TIF)

Figure S3 Photographs of posterior portion of the skull of *Mimoperadectes houdei* in (A) ventral (stereo photographs) and (C) left lateral views
(2.40 MB TIF)

Figure S4 Key to cranial measurements of *Mimoperadectes houdei*
(0.72 MB TIF)

Figure S5 Computer tomography reconstruction (CT) of right petrosal of *Mimoperadectes houdei* 482355 in ventral (A), dorsal (B), and lateral (C) views (scale above) and anatomical structures of the endocast of the right bony labyrinth in lateral (D) and dorsal (E) views (scale below). See Text S1 for abbreviations
(0.95 MB TIF)

Figure S6 Most parsimonious cladogram resulting from analysis of morphological data matrix. Numbers inside circles identify nodes. Of the numbers outside circles, those that are above indicate values of Bremer support and those that are below indicate values of jackknife support with 25% deletion of characters. Branches that display values of jackknife support below 73, received no support in a bootstrap search with 50% deletion of characters. Note: Mimo/peradectes stands for Mimoperadectes-Peradectes
(4.36 MB TIF)
