# Supplementary material for: Cranial Anatomy of the Earliest Marsupials and the Origin of Opossums
Source: PLoS One. 2009 Dec 16;4(12):e8278. doi: 10.1371/journal.pone.0008278 (PMC2789412; doi:10.1371/journal.pone.0008278)
Supplement: Text S1 — Anatomical description of the petrosal of Mimoperadectes houdei USNM 482355 (0.07 MB DOC) [file pone.0008278.s001.doc]

**Anatomical description of the petrosal of *Mimoperadectes houdei*** USNM 482355

Materials and methods for description of petrosal

The skull of *Mimoperadectes* was scanned with the micro-CT-Scanner RayScan 200 at the Fachhochschule Aalen, Germany. We used MIMICS software (® Materialise 2007, Release 11.1; license UMR 5143 CNRS-MNHN, Paris) to complete visualization, segmentation and 3D rendering. MIMICS is of great interest to deal with such a large amount of data sets, as those generated from X-Ray microtomography. We have used the MIMICS 64 bits version running on a Dell 690 Windows XP 64 workstation with 16 GB of RAM. MIMICS allows different types of measurement and segmentation to be performed. Regions of interest (i.e., petrosal bone and inner ear) can be selected with accuracy using threshold method to create segmentation masks. With this method, selections depend on a range of defined grey values, and not on manual outlining operations. 3D models have been calculated from segmentation masks and combined through Boolean operations. The software Cinema 4D (® Maxon 2007, Release 10; license UMR 5143 CNRS-MNHN, Paris) was used to improve the models by filling some gaps and smoothing the surface.

Measurements for *Mimoperadectes* are in millimetres and were taken directly on the 3D model with Mimics tools. The stapedial ratio was measured following the method by Segall [1] and the cochlear curvature, following the method by West [2].

**Anatomical Description of the middle- and inner-ear of *Mimoperadectes houdei* USNM 482355**

For descriptive purposes, the petrosal is generally divided in two parts: the pars cochlearis, enclosing the cochlear duct and saccule of the inner ear, and the pars canalicularis, housing the utricle and semicircular canals (e.g., [3]). In the following description we use the terminology of several relevant studies (e.g., [4-9]). The reconstruction of the major vessels and nerves associated with the petrosal is based on previous studies on extant mammals [6-8,10-16].

**Petrosal bone anatomy:** The two divisions of the petrosal are observable in the tympanic or ventral view (Figure S5); the pars cochlearis is represented by the teardrop-shaped and inflated promontorium and the flange projecting anteromedially from it, and the pars canalicularis by the bone lateral and posterior to the promontorium. The rounded fenestra cochleae is evident posteriorly, which was in life closed by the secondary tympanic membrane. Posterolaterally visible is the oval-shaped fenestra vestibuli, which was in life closed by the footplate of the stapes. The stapedial ratio of Segall [1], i.e., length/width of oval window or footplate, is 1.68, which is comparable to stapedial ratios of didelphids ([17]: table 3).

Anteromedial to the fenestra cochleae, the promontorium of *Mimoperadectes* exhibits a rostral tympanic process that forms well-developed ventrally projected wing on its whole length. Projecting anteromedially from the promontorium is a flat shelf of bone, the epitympanic wing (sensu [6]). The promontorium does not exhibit any sulci for either a stapedial artery or a transpromontorial internal carotid artery. These two features indicate a peculiar arterial pathway pattern that is typical for metatherian mammals (e.g., [18,19]).

The promontorium is laterally bordered by a tiny shelf of bone homologous to the eutherian tegmen tympani. Wible [8] described this structure as the tuberculum tympani for metatherians, which is actually the homologue of the eutherian tegmen tympani [20]. Between the tegmen tympani and the promontorium is a large and almost rounded aperture, the secondary facial foramen that transmits the main or hyomandibular branch of the facial nerve into the middle-ear space [7,9]. The main branch of the facial nerve runs posteroventrally in the sulcus facialis and exits the skull by the stylomastoid notch. Anteriorly to the secondary facial foramen is the large hiatus Fallopii that transmits the greater petrosal nerve or palatine ramus of the facial nerve to the posterior opening of the pterygoid canal.

Just anteromedial to the hiatus Fallopii and anterolaterally to the fenestra vestibuli is a pronounced fossa that was probably for attachment of the tensor tympani muscle, which is inserted into the manubrium of the malleus.

Just posterolateral to the secondary facial foramen is a large opening identified as the tympanic opening of the prootic canal. It corresponds with a lateral aperture visible in lateral view (Figure S5). The prootic canal of metatherians leads a vein from the prootic sinus to the rudimentary lateral head vein, which runs posteriorly to the geniculate ganglion of the facial nerve and to the otic capsule and passes through a groove lateral to the sulcus facialis.

The area of the pars canalicularis posterolateral to the promontorium and secondary facial foramen, and posteromedial to the tympanic aperture of the prootic canal shows a triangular depression, whose apex points posteriorly. The deepest part of this depression is the fossa incudis, which housed in life the crus breve of the incus. The fossa incudis is bordered posterolaterally by the squamosal, as in all extant metatherians. Antolateral to the fossa incudis is the shallower and broader epitympanic recess, which was in life dorsal to the tympanic membrane and housed the mallear-incudal articulation [21].

A low crista parotica posterolaterally borders the deep sulcus facialis and forms the medial wall of the fossa incudis. This crest supports a tiny protuberance, the tympanohyal (the ossified proximal segment of Reichert's cartilage), posterior to which is the stylomastoid notch by which the facial nerve probably left the middle ear.

The stylomastoid notch is bounded medially by a low ridge running posteromedially, the caudal tympanic process of petrosal (sensu [6]). The caudal tympanic process of petrosal extends medially from the stylomastoid notch to the jugular foramen (posterior lacerate foramen of Archer [4]), showing a slight decrease in height. The medial end of the caudal tympanic process of petrosal probably abuted the paracondylar process of the exoccipital bone.

Between the caudal tympanic process and the rear of the promontorium is a deep depression, whose narrower medial part is the postpromontorial tympanic sinus (sensu [7]), and whose broader and deeper lateral part is the fossa for the stapedius muscle.

The surface of the pars canalicularis posterolateral to the caudal tympanic process of petrosal is the mastoid exposure, the petrosal surface exposed on the occiput. On its anteriormost part the mastoid exhibits a tiny posteroventrally projected process which is likely to be the mastoid process of petrosal (sensu [4,7]), to which was attached the sternocleidomastoideus muscle.

On the posteriormost aspect of the mastoid exposure a light indentation may correspond to the trajectories of the diploetic vessels (see below), as seen in didelphid petrosal ([7]; S.L. pers.obs.).

The dorsal or endocranial view of the petrosal of *Mimoperadectes* is characterized by two dominant features (Figure S5). Anteromedially, the internal acoustic meatus for the facial and vestibulocochlear nerves lies on the roof of the pars cochlearis. Posteromedially the fossa subarcuata, which accommodated the paraflocculus of the cerebellum, forms a very large fossa, relative to the size of the internal acoustic meatus. The fossa subarcuata is conical and deep and is bounded by the three semicircular canals (see below).

On the dorsoposterolateral corner of the pars canalicularis, behind the crus commune and posterior semicircular canal, is the narrow groove for the sigmoid sinus. This sinus is the posterodorsal branch of the transverse sinus (the anteroventral branch being the prootic sinus and sphenoparietal emissary vein) [22].

A broad sulcus running along the dorsomedial edge of the promontorium received the inferior petrosal sinus, which originated at the cavernous sinus around the hypophysis and left the skull via its own foramen or via the jugular foramen (see [8]).

The aqueductus vestibuli for passage of the endolymphatic duct is posteromedial to the fossa subarcuata and near the crus commune. The aqueductus cochleae for passage of the perilymphatic duct, is posteromedial to the internal acoustic meatus, but hidden in dorsal view by a bony bar behind the foramen acusticum inferius. The aqueductus cochleae opens into the jugular foramen, which transmits cranial nerves (presumably the glossopharyngeal, vagus, and accessory nerves as in didelphids [8]).

In lateral view, the lateral aperture of the prootic canal is evident just posterolateral to the secondary facial foramen (Figure S5). The lateral aperture of the prootic canal receives a small vein from the prootic sinus, the sulcus of which being dorsolateraly directed. This small vein exits the prootic canal via the tympanic aperture as the lateral head vein. In extant adult marsupials, the development of the sphenoparietal emissary vein causes a reduction of the tympanic portion of the lateral head vein [7]. The lateral head vein and the prootic canal are retained in adult monotremes [16], the eutherian *Prokennalestes* [23] and “zhelestid” eutherians [24], the metatherians *Deltatheridium*, *Didelphodon*, *Pediomys* [19,23], *Pucadelphys*, *Andinodelphys* [25,26], and some extant marsupials (didelphids, some caenolestids, peramelinans, and dasyuromorphians [7,27]).

A tiny sulcus starts just posteromedially to the lateral aperture of the prootic canal and directs posteromedially along the part of the pars canalicularis covered by the squamosal. This groove may have transmitted the diploetic vessels (the vena diploëtica magna and arteria diploëtica magna [7,15]) from the area dorsal to the postglenoid foramen to the posttemporal foramen (sensu [7,28]). A notch on the posteriormost part of the pars canalicularis may be identified as the medial edge of the posttemporal foramen, which is evident between the petrosal and squamosal when we consider the entire basicranium. Such a condition was described for didelphids [7,8,27].

# Inner ear anatomy: The osseous labyrinth consists of three parts: the cochlea, which contained the cochlear duct; the vestibule, which contained the utricle and saccule; and the semicircular canals, which contained the semicircular ducts (Figure S5).

The most prominent feature of the osseous labyrinth is the cochlea, which is a coiled, broad, hollow tube of uniform diameter. It occupies the majority of the available space in the pars cochlearis with little room to spare. The cochlea has 2.1 spiral turns, almost the same as reported for *Didelphis* (2.5 [29,30]) and *Caluromys* (2.4 [31]). It is noteworthy *Herpetotherium* has been reported as having a low number of cochlear turns (1.6 [32]).

Just posteromedial to the fenestra cochleae is the long, narrow cochlear aqueduct of the perilymphatic duct.

The connection between the cochlea and vestibule is at the posteromedial aspect of the pars cochlearis. The vestibule communicates with the cochlea anteriorly and the semicircular canals posteriorly. It is an irregular, oval, central space, which is joined distally by the ampullae of the three semicircular canals. As in most mammals, the semicircular canals join the utricle through five openings: one for the crus commune, the others for the medial entrance to the lateral semicircular canal and the three ampullae (anterior, lateral, posterior). The anterior ampulla is dorsolateral to the vestibule, the lateral ampulla ventrolateral, and the posterior ampulla ventromedial. The lateral semicircular canal lies in a nearly horizontal plane in the floor of the fossa subarcuata. The posterior semicircular canal is in a nearly vertical plane in the medial wall of the fossa subarcuata; it joins the anterior canal in the crus commune dorsally and the lateral canal ventrally. The posterior arm of the lateral semicircular canal and the inferior arm of the posterior semicircular canal build a second crus commune, a condition found in *Caluromys*, *Monodelphis*, *Dasyurus*, *Isoodon*, *Herpetotherium* from the Oligocene of Wyoming [32], and in a palaeothentid caenolestoid from the early Miocene of Patagonia [31] among metatherians.

**Abbreviations used in Figure S5**: ant., anterior; dors., dorsal; med., medial; aa, anterior ampulla; ac, aqueductus cochleae; adm, arteria diploëtica magna; asc, anterior semicircular canal; av, aqueductus vestibuli; cc, crus commune; coaq, cochlear aqueduct; cp, crista parotica; co, cochlear duct; cr, crista petrosa; ctpp, caudal tympanic process of petrosal; er, epitympanic recess; fai, foramen acousticum inferius; fas, foramen acousticum superius; fc, fenestra cochleae; fi, fossa incudis; fn, facial nerve; fsa, fossa subarcuata; fv, fenestra vestibuli; gg, geniculate ganglion; gpn, greater petrosal nerve; hF, hiatus Fallopii; iam, internal auditory meatus; ips, inferior petrosal sinus; la, lateral ampulla; lhv, lateral head vein; lsc, lateral semicircular canal; mp, mastoid process; pa, posterior ampulla; pc (lata), prootic canal lateral aperture; pc (tympa), prootic canal tympanic aperture; pcv, prootic canal vein; pfc, prefacial commissure; pr, promontorium; ps, prootic sinus; psc, posterior semicircular canal; ptn, posttemporal notch; pts, posttemporal sulcus; rtpp, rostral tympanic process of petrosal; scc, second crus commune; sf, stapedius fossa; sff, secondary facial foramen; sips, sulcus for inferior petrosal sinus; smn, stylomastoid notch; spev, sphenoparietal emissary vein; sps, sulcus for prootic sinus; ss, sigmoid sinus; sss, sulcus for sigmoid sinus; th, tympanohyal; ts, transverse sinus; ttf, tensor tympani fossa; vdm, vena diploëtica magna; ve, vestibule.

# References

1. Segall W (1970) Morphological parallelisms of the bulla and auditory ossicles in some insectivores and marsupials. Fieldiana Zool 51:169-205.

2. West CD (1985) The relationship of the spiral turns of the cochlea and the length of the basilar-membrane to the range of audible frequencies in ground dwelling mammals. J Acoust Soc Am 77:1091-1101.

3. Voit M (1909) Das Primordialcranium des Kaninchens unter Berücksichtigung der Deckknochen. Ein Beitrag zur Morphologie des Säugetierschädels. Anat Hefte 38:425-616.

4. Archer M (1976) The basicranial region of marsupicarnivores (Marsupialia), interrelationships of carnivorous mammals, and affinities of the insectivorous marsupial peramelids. Zool J Linn Soc 59:217-322.

5. Luo Z-X, Kielan-Jaworowska Z, Cifelli RL (2002) In quest for a phylogeny of Mesozoic mammals. Acta Palaeontol Pol 47:1-78.

6. MacPhee RDE (1981) Auditory regions of primates and eutherian insectivores: morphology, ontogeny, and character analysis. Contrib Primatol 18:1-282.

7. Wible JR (1990) Petrosals of Late Cretaceous marsupials from North America, and a cladistic analysis of the petrosal in therian mammals. J Vertebr Paleontol 10:183-205.

8. Wible JR (2003) On the cranial osteology of the short-tailed opossum *Monodelphis brevicaudata* (Didelphidae, Marsupialia). Ann Carnegie Mus 72:137-202.

9. Wible JR, Hopson JA (1993) Basicranial evidence for early mammal phylogeny. In: Szalay FS, Novacek MJ, McKenna MC, editors. Mammal Phylogeny: Mesozoic differentiation, multituberculates, monotremes, early therians, and marsupials. New York: Springer-Verlag. pp. 45-62.

10. Novacek MJ (1986) The skull of leptictid insectivorans and the higher-level classification of eutherian mammals. Bull Am Mus Nat Hist 183:1-111.

11. Novacek MJ (1993) Patterns of diversity in the mammalian skull. In: Hanken J, Hull B, editors. The skull: patterns of structural and systematic diversity. Chicago: University of Chicago Press. pp. 438-545.

12. Rougier G, Wible J, Hopson J (1992) Reconstruction of the cranial vessels in the early Cretaceous mammal *Vincelestes neuquenianus*: Implications for the evolution of the mammalian cranial vascular system. J Vertebr Paleontol 12:188-216.

13. Wible JR (1984) The ontogeny and phylogeny of the mammalian cranial arterial pattern (internal carotid artery). PhD Thesis. Duke University, Durham, North Carolina.

14. Wible JR (1986) Transformations in the extracranial course of the internal carotid artery in mammalian phylogeny. J Vertebr Paleontol 6:313-325.

15. Wible JR (1987) The eutherian stapedial artery: character analysis and implications for supraordinal relationships. Zool J Linn Soc 91:107-135.

16. Wible JR, Hopson JA (1995) Homologies of the prootic canal in mammals and non-mammalian cynodonts. J Vertebr Paleontol 15:331-356.

17. Horovitz I, Ladeveze S, Argot C, Macrini TE, Martin T, et al. (2008) The anatomy of *Herpetotherium* cf. *fugax* COPE, 1873, a metatherian from the Oligocene of North America. Palaeontogr Abt A 284:109-141.

18. Rougier GW, Wible JR (2006) Major changes in the ear region and basicranium of early mammals. In: Carrano MT, Gaudin TJ, Blob RW, Wible JR, editors. Amniote paleobiology: Phylogenetic and functional perpectives on the evolution of mammals, birds and reptiles. Chicago: University of Chicago Press. pp. 269-311.

19. Rougier GW, Wible JR, Novacek MJ (1998) Implications of *Deltatheridium* specimens for early marsupial history. Nature 396:459-463.

20. Kuhn HJ, Zeller U (1987) The cavum epiptericum in monotremes and therian mammals. In: Kuhn HJ, Zeller U, editors. Morphogenesis of the mammalian skull. Hamburg: Verlag Paul Varey. pp. 51-70.

21. Van der Klaaw C (1931) The auditory bulla in some fossil mammals, with a general introduction to this region of the skull. Bull Am Mus Nat Hist 62:1-352.

22. Dom R, Fisher BL, Martin GF (1970) The venous system of the head and neck of the opossum (*Didelphis virginiana*). J Morphol 132:487-496.

23. Wible JR, Rougier GW, Novacek MJ, McKenna MC (2001) Earliest eutherian ear region: a petrosal referred to *Prokennalestes* from the Early Cretaceous of Mongolia. Am Mus Novit 3322:1-44.

24. Ekdale EG, Archibald JD, Averianov AO (2004) Petrosal bones of placental mammals from the Late Cretaceous of Uzbekistan. Acta Palaeontol Pol 49:161-176.

25. Ladevèze S, Muizon C de (2007) The auditory region of early Paleocene Pucadelphydae (Mammalia, Metatheria) from Tiupampa, Bolivia, with phylogenetic implications. Palaeontology 50:1123-1154.

26. Marshall LG, Muizon C de, Sigogneau-Russell D (1995) *Pucadelphys andinus* (Marsupialia, Mammalia) from the early Paleocene of Bolivia. Mém Mus natl Hist nat 165:1-164.

27. Sánchez-Villagra MR, Wible JR (2002) Patterns of evolutionary transformation in the petrosal bone and some basicranial features in marsupial mammals, with special reference to didelphids. J Zool Syst Evol Res 40:26-45.

28. Wible JR, Rougier GW, Novacek MJ, McKenna MC, Dashzeveg D (1995) A mammalian petrosal from the early Cretaceous of Mongolia: Implications for the evolution of the ear region and mammaliamorph interrelationships. Am Mus Novit 3149:1-19.

29. Fernández C, Schmidt RS (1963) The opossum ear and evolution of coiled cochlea. J Comp Neurol 121:151-159.

30. McCrady E (1938) The embryology of the opossum. Am Anat Mem 16:1-233.

31. Sánchez-Villagra M, Schmelzle T (2007) Anatomy and development of the bony inner ear in the woolly opossum, *Caluromys philander* (Didelphimorphia, Marsupialia). Mastozool Neotrop 14:53-60.

32. Sánchez-Villagra MR, Ladevèze S, Horovitz I, Argot C, Hooker JJ, et al. (2007) Exceptionally preserved North American Paleogene metatherians: adaptations and discovery of a major gap in the opossum fossil record. Biol Lett 3:318-322.
