# Supplementary material for: Cranial Anatomy of the Earliest Marsupials and the Origin of Opossums
Source: PLoS One. 2009 Dec 16;4(12):e8278. doi: 10.1371/journal.pone.0008278 (PMC2789412; doi:10.1371/journal.pone.0008278)
Supplement: Text S2 — List of characters and phylogenetic analysis (0.22 MB DOC) [file pone.0008278.s002.doc]

list of characters and phylogenetic analysis 

The list of characters is based on Horovitz and Sánchez-Villagra [1], with certain modifications/additions introduced by Sánchez-Villagra et al. [2] and Horovitz et al. [3](2008: 126), and some new changes as detailed below.
The data matrix includes a new terminal taxon labelled Mimoperadectes-Peradectes consisting mostly of dentition and cranium of Mimoperadectes houdei USNM 482355 plus some additional codings from the dentition Mimoperadectes labrus and Peradectes sps., and a very limited number of postcranial characters from a Peradectes-like species from the Middle Eocene of Messel, Germany, which may belong to this same genus or alternatively may be closely related to it. The specimens and/or literature examined were:

Mimoperadectes labrus: Descriptions and illustrations from Bown and Rose [4], Strait [5] and Gingerich and Smith [6]. 

Peradectes: Descriptions and illustrations of dental specimens by Clemens [7], Crochet [8], Clemens and Lillegraven [9] and Fox [10]; postcranium of HLMD-Me-17001 and HLMD-Me-8035 (HLMD-Me: Hessisches Landesmuseum Darmstadt, Messel collection, Germany). Due to preservation, we were able to code only three characters for the postcranium of these specimens: 30 (HS31), 47 (HS48), and 86 (HS89).

Codings for Herpetotherium were expanded and it was possible to score additional characters for the forelimb of Herpetotherium cf. fugax based on the new specimens SMF 2000/168 and 169 (Figure 4). Coding for ch. 21 (HS22) also added. 

Note: Characters 1-218 are based on Horovitz and Sánchez-Villagra ([1]; HS refers to the character numbering in that reference], whereas characters 219-259 are based on Ladevèze [11] and Ladevèze and Muizon ([12]; LA refers to numberings in these references). See these references and Sánchez-Villagra et al. [2] and Horovitz et al. [3] for more details on character definitions.
Characters treated as ordered are indicated below with “*”.
The following corrections are noted: in character HS170 (number 155 in Sánchez-Villagra et al. (2], 167 here], the definitions of character states “0” and “1” were inverted, in other words “0” should stand for a reduced or absent upper canine and “1” for a canine that is caniniform or premolariform; in character HS172 (number 157 in Sánchez-Villagra et al. [2], 169 here], the coding for Herpetotherium is “1”, meaning the staggering in a lower incisor (after Hershkovitz [13]]; in character LA22 (number 226 in Sánchez-Villagra et al. [2], 240 here] codings for Didelphis and Monodelphis should be “1” which stands for a small, slanted and nodelike mastoid tympanic process; all multiple entries should be enclosed in “( )”, indicating polymorphic or heterogeneous taxa (although several entries were inadvertently enclosed in “{}” before).

1. (HS 1) Atlantal foramen: 0 = absent; 1 = present.
2. (HS 3) Atlas transverse foramen: 0 = absent; 1 = present.
3. (HS 4) Atlas, posterior extent of transverse process: 0 = absent; 1 = present.
We coded this character as "present" when the transverse processes extend caudally beyond the caudal facets for the axis.
4. (HS 5) Atlas, cranial facets shape*: 0 = only concave; 1 = dorsal edge curved; 2 = dorsal edge envelops the occipital condyle medially.
We coded "0" when the surface is more or less equally concave along its entirety (same, even curvature), "1" when the dorsal end of the facet is more curved (concave) than the rest of the facet, covering the condyles dorsally, and "2" when the dorsal end of the facet curves even further, enveloping the condyles reaching their medial side. To further clarify, the dorsal end of these facets coded "2" faces almost laterally or laterally.
5. (HS 6) Atlas, transverse process ventral to atlantal foramen groove: 0 = absent; 1 = present.
We coded "1" those taxa where the transverse process extends anteriorly reaching the area ventral to the atlantal foramen, or if the latter was missing, ventral to the groove for the vertebral artery. We coded "0" those taxa where the transverse process does not reach so far anteriorly.
6. (HS 7) Atlas ventral arches: 0 = open; 1 = complete.
We mean by "complete" cases in which there was no space between the two arches ventrally; in cases where it is complete, presence or absence of an intercentrum is not considered here.
7. (HS 8) Atlas intercentrum*: 0 = absent; 1 = present with no fusion; 2 = present, fused. 
Only two character states are recorded in matrix, therefore * does not apply.
If there are no sutures visible on the ventral arch and there is no information from histological sections as to the presence of an intercentrum or not, the character is coded as missing.
8. (HS 9) Axis transverse foramen: 0 = open; 1 = enclosed.
9. (HS 10) Axis posterior spinous process extension: 0 = absent; 1 = present.
We coded "1" when the neural spine extends posteriorly beyond the vertebral arches.
10. (HS 11) Axis extra pair of transverse processes on the ventral surface of the body: 0 = absent; 1 = present.
11. (HS 12) Axis anterior facets (prezygapophyses) and dens connection*: 0 = not linked; 1 = linked; 2 = facets extend ventral to the dens.
"Linked" refers to the condition in which there is no clear border between the articular facets and the dens, but rather a continuous surface.
12. (HS 13) Suture between rib and axis is visible: 0 = no; 1 = suture visible.
13. (HS 14) Axis-C3-C4 fusion*: 0 = no fusion; 1 = axis and C3 fused; 2 = axis-C3-C4 fused.
14. (HS 15) C3-C4 ventral sagittal process: 0 = absent; 1 = present.
15. (HS 16) C5 transverse process displays two heads: 0 = absent; 1 = present.
16. (HS 17) C5 transverse process heads overlap transversally: 0 = absent; 1 = present.
17. (HS 18) C5 and T1 body length: 0 = subequal or C5 longer than T1; 1 = C5 shorter than T1.
The anteroposterior length of vertebrae C5 and T1 was measured in ventral view and the ratio C5/T1 was calculated for each taxon. Taxa with values equal to or larger than 0.9 were coded as “0” and those equal or smaller than 0.8 were coded as “1”.
18. (HS 19) C6 transverse process posterior extension: 0 = absent; 1 = present.
We coded "present" when the transverse process extends posteriorly beyond the articulation between the bodies of C6 and C7.
19. (HS 20) C6 spinous process*: 0 = absent; 1 = protuberance; 2 = lamina.
20. (HS 21) C7 transverse foramen*: 0 = absent; 1 = incipient; 2 = present.
21. (HS 22) Articulation among cervical vertebrae: 0 = only bodies articulate; 1 = prezygapophyses and postzygapophyses in addition to bodies articulate.
22. (HS 23) Articulation between C4-C5 bodies type: 0 = flat; 1 = saddle.
We coded “saddle’ when the anterior side of each body embraces the posterior side of the previous (anterior) body.
23. (HS 24) T1 transverse process: 0 = absent; 1 = present.
24. (HS 25) T1 transverse process level: 0 = level with prezygapophysis; 1 = lower than prezygapophysis.
25. (HS 26) First thoracic vertebra with a tall spinous process (relative to other vertebrae): 0 = T1; 1 = T2; 2 = T3.
26. (HS 27) First thoracic vertebrae with prezygapophysis facing laterally*: 0 = T1; 1 = T2; 2 = T3.
27. (HS 28) Thoracic vertebrae, intervertebral foramen enclosed: 0 = absent; 1 = present.
28. (HS 29) Last lumbar vertebra, foramen on dorsal arch: 0 = absent; 1 = present.
This foramen is often present in other lumbar vertebrae as well. We note that the available lumbar vertebra of Andinodelphys has no foramen on its dorsal arch, but its serial homology is unclear, so we prefer to code this character as “unknown” for Andinodelphys.
29. (HS 30) Metapophyses in third lumbar vertebra anterior to last: 0 = absent; 1 = anterior; 2 = dorsal 
30. (HS 31) Caudal vertebrae, count: 0 = 9 or fewer; 1 = 11 or more.
31. (HS 32) Caudal vertebrae, hyperexpanded chevrons: 0 = absent; 1 = present.
The character state "present" refers to the condition in which the chevron bones are as wide as the vertebral bodies. Those taxa with condition "1" also display proximal and distal expansions.
32. (HS 33) Prehensile tail: 0 = absent; 1= present.
33. (HS 34) Sternal cartilages, ossification (= sternal ribs): 0 = absent; 1 = present.
34. (HS 35) Ribs, articulation with sternum: 0 = with single sternebra; 1 = with two sternebrae.
35. (HS 36) Coracoid process*: 0 = separate and large; 1 = hook-like process; 2 = small process; 3 = small protuberance or absent.
36. (HS 37) Ventral extension of acromion: 0 = ventral (beyond) glenoid; 1 = does not extend beyond level of glenoid.
37. (HS 38) Crest on medial aspect of scapula, near the caudal border: 0 = absent; 1 = present.
38. (HS 39) Separate large interclavicle: 0 = absent; 1 = present.
39. (HS 40) Infraspinous fossa width*: 0 = less than 1/4 its length; 1 = between 1/2 and 1/4 its length; 2 = more than 1/2 its length.
The width of the infraspinous fossa was measured as maximum width perpendicular to scapular spine.
40. (HS 41) Scapular spine width at neck level*: 0 = narrower than infraspinous fossa; 1 = subequal; 2 = wider than infraspinous fossa.
41. (HS 42) Infraspinous/supraspinous fossa width at neck level*: 0 = infraspinous fossa narrower; 1 = subequal; 2 = supraspinous fossa narrower.
42. (HS 43) Humerus, medial relief for teres major m.: 0 = absent; 1 = present.
The presence of a rugosity, prominence, or ridge was scored as "1." The condition in Phalanger maculatus is absent, that in P. celebensis (AMNH-107999, 108000) is present.
43. (HS 44) Humerus, lateral ridge*: 0 = absent; 1 = ridge or crest; 2 = massive crest continuous with deltopectoral crest.
44. (HS 45) Humerus, capitulum for radius: 0 = spherical; 1 = cylindrical.
45. (HS 46) Humerus, entepicondylar foramen: 0 = absent; 1 = present.
46. (HS 47) Humerus, olecranon fossa or foramen*: 0 = absent; 1 = small fossa; 2 = large fossa; 3 = foramen.
This character was coded as "1" when a fossa is present but when its boundaries are not delimited proximally or it was narrower than the distal articular surface of the humerus in distal view. Coding “2” was assigned to fossae that fulfill both conditions (they are delimited around their whole perimeter and were as wide as the distal articular surface of the humerus in posterior view).
47. (HS 48) Humerus, laminar supinator crest: 0 = absent; 1 = present.
48. (HS 49) Humerus, greater tuberosity height relative to humeral head height: 0 = equal or greater tuberosity is lower; 1 = greater tuberosity is higher.
49. (HS 50) Humerus, extension of the deltoid crest: 0 = restricted to proximal half of humerus; 1 = reaches distal half.
50. (HS 51) Humerus trochlea: 0 = absent; 1 = present.
51. (HS 52) Humerus, proximal extension of capitulum and trochlea*: 0= longer proximal extension of trochlea; 1 = equal; 2 = longer proximal extension of capitulum. 
Only character states 0 and 1 recorded in matrix, therefore * does not apply.
52. (HS 53) Humerus, medial epicondyle size: 0 = small; 1 = large.
We measured the mediolateral size of the medial epicondyle and divided it by that of the proximal epiphysis of the humerus. Values equal to 0.44 or larger were coded as "large," and values equal to 0.4 or smaller were coded as "small." The medial epicondyle is small in Didelphis marsupialis but large in D. virginiana.

53. (HS 54) Humerus distal end size: 0 = small; 1 = large.
We computed a quotient between the mediolateral size of the distal epiphysis of the humerus excluding the medial epicondyle and the proximal epiphysis. Quotients equal to 0.85 or smaller were considered "small" and those equal to 0.9 or larger were considered "large."
54. (HS 55) Humerus, head shape: 0 = symmetrical/ovoid; 1 = mediolaterally compressed.
45. (HS 56) Humerus, capitulum lateral extension: 0 = absent; 1 = present.
An extension is recorded when the articular surface extends beyond a rounded or cylindrical capitulum, in the shape of a raised edge, or a groove and an additional shelf.
56. (HS 57) Humerus, shaft curvature: 0 = straight; 1 = curved.
57. (HS 58) Ulna, proximal epiphysis, distal most portion of articular surface for humerus: 0 = ulnar area is less than 1/2 of radial one; 1 = more.
This portion of the ulnar articular surface was coded as “0” when its area was less than half the size of the radial proximal articular surface. If it was larger, it was coded as "1."
58. (HS 59) Ulna, coronoid process: 0 = absent; 1 = present.
59. (HS 60) Distal process of ulna: 0 = spherical, contributing to a ball and socket articulation; 1 = nonspherical.
60. (HS 61) Radius, articular facet for humerus: 0 = circular; 1 = antero-posteriorly compressed.
61. (HS 62) Lunate*: 0 = absent or fused to other elements; 1 = very small; 2 = relatively large, with contacts with other carpals.
62. (HS 63) Prepollex: 0 = absent; 1 = present.
63. (HS 64) Distolateral process of scaphoid separates dorsally lunate from magnum*: 0 = absent; 1 = present; 2 = present, separates dorsally lunate from magnum.
64. (HS 65) Proximal aspect of magnum: 0 = single head; 1 = saddle-shape articulation with lunate.
65. (HS 66) Magnum, facet with MC III: 0 = flat, convex or keeled; 1 = fully concave.
66. (HS 67) Trapezium, number of distal heads: 0 = one head; 1 = two heads.
67. (HS 68) Plane of the articular surface trapezium–Mc I in relation to other carpal–metacarpal articulations: 0 = not parallel; 1 = somewhat parallel.
68. (HS 69) Both MC I and MC V are hyper-reduced in relation to other metacarpals: 0 = hyperreduction; 1 = no hyperreduction.
69. (HS 70) Magnum and unciform proximal extension: 0 = similarly proximal; 1 = unciform more proximal.
70. (HS 71) Tuberosity for rectus femoris m.: 0 = no relief; 1 = protuberance; 2 = depression.
71. (HS 72) Relative size of obturator foramen: 0 = smaller or equal to acetabulum; 1 = larger than acetabulum.
72. (HS 73) Hyperdevelopment of iliopubic process: 0 = absent; 1 = present.
73. (HS 75) Pelvis, acetabular notch: 0 = absent; 1 = present.
74. (HS 76) Epipubic bones: 0 = absent; 1 = present.
75. (HS 77) Epipubic bones proximal size: 0 = short; 1 = long.
The coding "0" was applied to cases where the contact extent is equal to or less than half the distance that between the pubic symphysis and the point at the anterior edge of the pelvis which is level with the middle of the acetabulum; the coding "0" was applied to cases where the contact is larger.
76. (HS 78) Mediolateral constriction in greater trochanter of femur: 0 = absent; 1 = present.
77. (HS 79) Relative height of greater trochanter / femoral head: 0 = greater trochanter is lower or equal; 1 = higher.
To code this character, the femoral capitula were positioned on a horizontal surface (rather than orienting the femoral shaft on a perfectly vertical plane).
78. (HS 80) Femur, lesser trochanter: 0 = absent; 1 = present. 
A small relief in the area of the lesser trochanter was coded as “absent” for this character.
79. (HS 81) Fibula proximal medial accessory shelf: 0 = absent; 1 = present.
In some cases the fibula articulates with the tibia through a facet located on a process or shelf that projects medially from the medial surface of the proximal fibula. In others, this projection is missing and the fibula displays a facet located directly on the proximal surface of the fibula.
80. (HS 82) Fibula proximal posterior extension beyond the area of contact with the tibia: 0 = does not extend posteriorly; 1 = extends.
The fibula may be very wide anteroposteriorly in its proximal end. In cases where it is very wide, it extends posteriorly to the facet of contact with the tibia.
81. (HS 83) Fibula lateral distal process: 0 = absent; 1 = present.
82. (HS 84) Articulation between femur and fibula: 0 = absent; 1 = present.
Asiatherium is coded “1” contra Luo et al. [14]. Although the knee area in available specimens is in poor condition, there is evidence of a fibula–femur contact (see also Szalay and Trofimov [15]).
83. (HS 85) Process on fibula proximal end extends proximally beyond knee: 0 = absent; 1 = present.
84. (HS 86) Sesamoids in articular area between tibia, fibula, and astragalus (or UAJ, upper ankle joint)*: 0 = absent; 1 = one sesamoid present; 2 = two sesamoids present.
85. (HS 88) Tibia medial malleolus: 0 = absent; 1 = present.
Asiatherium is coded as missing (“?”) contra Luo et al. [14], who coded it as “weak”. The distal end of the tibia is somewhat crushed and it is not possible to infer the presence of a malleolus.
86. (HS 89) Tibia length relative to femur length: 0 = tibia shorter than femur; 1 = tibia longer or equal to femur.
87. (HS 90) Tibia proximal dimensions*: 0 = larger mediolaterally than anteroposteriorly; 1 = equal; 2 = smaller mediolaterally than anteroposteriorly.
88. (HS 91) Tibia posterior shelf*: 0 = absent; 1 = present but does not extend posteriorly beyond the medial astragalotibial facet; 2 = present and extends posteriorly beyond the medial astragalotibial facet.
The “tibia posterior shelf” is something close in definition to the “Atip” (posterior astragalotibial facet) of Szalay ([16]: e.g. fig. 4.4, p. 100). However, Szalay did not really define its limits. Hence, we name this character “tibia, posterior shelf” and define the conditions we were able to observe in the specimens ourselves. This “shelf” is present and displays condition “2” in the illustrations of Metachirus and Phalanger of figure 4.4 of Szalay, for example, where we coincide with Szalay’s ATip. Note that in both of these cases there is a notch between the ATip and the posterior area of the ATim (medial astragalotibial facet). In both Metachirus and Phalanger, the structure marked as ATip protrudes posteriorly beyond the ATim. In the same figure, Tupaia and Hypsiprymnodon have no posterior tibial shelf (our condition “0”) and no ATip. In addition, there is no notch lateral to the posterior area of the ATim. There are no examples of our third case, condition “1”, in Szalay [16] but we can take Metachirus from figure 4.4 [16] and transform it to fit condition “1” for the sake of our explanation: if we preserve the notch but remove the ATip and leave the area lateral to the notch (=ATil) ending posteriorly at the same level as the ATim, we have condition =1. We are not sure if Szalay would consider ATip to be present in such a case, but we define the “tibia posterior shelf” as present in such cases and we consider this condition “1” of a “tibia posterior shelf” level with the ATim with a notch in between, to be intermediate between “2 = present and protruding” and “0 = absent”.
89. (HS 92) Tibia, distal articulation type: 0 = sagittal; 1 = spiral.
90. (HS 93) Tibia, posterior shelf articulation: 0 = not articular; 1 = articular.
91. (HS 94) Astragalus, angle between medial and lateral facets for tibia*: 0 = 90 degrees; 1 = intermediate; 2 = 180 degrees.
92. (HS 95) Astragalonavicular facet extends on medial side of head: 0 = absent; 1 = medial.
93. (HS 96) Astragalonavicular facet extends on ventromedial area of head: 0 = absent; 1 = present.
94. (HS 97) Astragalus, dimensions of facet for navicular in distal view: 0 = transversely wider; 1 = dorsoventrally wider.
95. (HS 98) Astragalar medial plantar tuberosity (ampt) visibility in dorsal view: 0 = not visible; 1 = visible.
96. (HS 99) Astragalus, angle between facet for fibula and lateral facet for tibia*: 0 = 180 degrees; 1 = intermediate; 2 = 90 degrees.
In contrast to HS 99, character state 0 is now defined as: 0 = 180 or more degrees.
97. (HS 100) Astragalar neck: 0 = absent; 1 = present.
98. (HS 101) Astragalus, relative width of head and neck: 0 = neck narrower or as wide as head; 1 = neck wider than head.
99. (HS 102) Astragalar sustentacular facet medial extent: 0 = does not reach medial edge of neck; 1 = reaches medial edge of neck.
100. (HS 103) Astragalonavicular facet position relative to facets for tibia: 0 = anterior to facets for tibia; 1 = medial relative to facets for tibia.
101. (HS 104) Astragalar canal: 0 = absent; 1 = present.
102. (modified HS 105) Posterior astragalocalcaneal facet major dimension orientation: 0 = straight; 1 = posteromedial to anterolateral.
103. (HS 106) Posterior astragalocalcaneal facet position in ventral view: 0 = extending up to posterior edge of astragalus; 1 = subterminal.
The state “1” refers to the condition in which the facet does not extend up to the posterior end of the astragalus but ends a little more anteriorly.
104. (HS 107) Astragalus, ridge between medial and lateral astragalotibial facets: 0 = absent; 1 = present.
105. (HS 108) Astragalus, ridge between lateral astragalotibial and astragalofibular facets: 0 = absent; 1 = present.
106. (HS 109) Medial astragalotibial facet, posterior extent: 0 = does not reach posterior edge of lateral astragalotibial facet, 1= equal in anteroposterior length as lateral astragalotibial facet.
107. (HS 110) Astragalonavicular facet connection with sustentacular facet: 0 = absent; 1 = present.
108. (HS 111) Calcaneal sustentacular facet on sustentaculum*: 0 = no sustentaculum (facet is located in calcaneal body); 1 = on sustentaculum; 2 = facet is located above level of sustentaculum, which becomes the medial process.
109. (HS 112) Calcaneal sustentaculum position: 0 = subterminal; 1 = on anterior end.
110. (HS 113) Ectal (or posterior calcaneoastragalar facet) longest dimension*: 0 = anteromedial to posterolateral; 1 = straight; 2 = posteromedial to anterolateral.
111. (HS 114) Ectal facet longest axis when straight: 0 = sagittally longer; 1 = transversely longer.
When the previous character is scored as "1 = straight," this condition was considered here for scoring in the two alternative varieties. All other conditions of the previous character were inapplicable here.   
112. (HS 115) Calcaneal anterior peroneal tubercle: 0 = absent; 1 = present.
113. (HS 116) Calcaneal anterior peroneal tubercle shape*: 0 = protuberance; 1 = laminar; 2 = process.
114. (HS 117) Calcaneal anterior peroneal tubercle position*: 0 = protruding anteriorly beyond calcaneocuboid facet; 1 = anterior, non-protruding; 2 = at a distance from anterior end of calcaneum.
The anterior edge of the anterior peroneal tubercle in some cases is located at a short distance from the anterior end of the lateral side of the calcaneum (condition “2”), in other cases its anterior edge is level with the calcaneocuboid facet (condition “1”), and finally, it may protrude anteriorly, with its tip extending anteriorly beyond the level of the calcaneocuboid facet (condition “0”).
115. (HS 118) Calcaneal sustentacular facet mesiolateral orientation: 0 = medial; 1 = dorsal. 
The sustentacular facet was scored as medial when at least the anterior area is directed medially, even if more posteriorly it curves to facing somewhat more dorsally.
116. (HS 119) Calcaneal sustentacular facet anteroposterior orientation: 0 = dorsal; 1 = 45 degrees dorsoanteriorly. This character was only coded for taxa having ch. HS 118 (1).
117. (HS 120) Calcaneal sustentacular facet anteroposterior convexity: 0 = concave or flat; 1 = convex.
118. (HS 121) Calcaneal sustentacular facet posteriorly convex: 0 = absent; 1 = present. 
Coded as “-” to avoid redundancy with previous character for taxa coded as 117 (1).
119. (HS 122) Calcaneal plantar tubercle: 0 = absent; 1 = present.
120. (HS 123) Calcaneal sustentacular facet and posterior calcaneoastragalar facets merging*: 0 = separate; 1 = with narrow connection; 2 = merged.
121. (HS 124) Calcaneal sustentacular facet anterior edge: 0 = anterior to posterior facet; 1 = equal or posterior to posterior facet.
122. (HS 125) Calcaneal facet for fibula: 0 = absent; 1 = present.
123. (HS 126) Calcaneal facet for fibula orientation*: 0 = lateral; 1 = dorsal; 2 = dorsal only posteriorly. 
Although all three character states were recorded in the matrix, character state “2” was inadvertently omitted in the original character description [17].
124. (HS 127) Calcaneal notch for cuboid pivot: 0 = absent; 1 = present.
125. (HS 128) Calcaneum sustentacular facet reaches anterior end: 0 = absent; 1 = present.
126. (HS 129) Calcaneum accessory facet anterior to sustentacular facet: 0 = absent 1 = present.
127. (HS 130) Cuboid medial plantar process forms groove: 0 = absent; 1 = present.
The cuboid medial plantar process in some cases displays a hook-like shape, forming an almost enclosed canal for the tendon of the peroneus longus m. [16].
128. (HS 131) Cuboidcalcaneal facet: 0 = mostly convex or flat; 1 = concave anteroposteriorly; 2 = two shelves, medial more proximal; 3 = two concavities.
129. (HS 132) Cuboidcalcaneal facet angle between proximal and distal facet areas*: 0 = no angle; 1 = angle present and small, dorsal area narrower than proximal area; 2 = angle present and almost straight, dorsal area wider than width of proximal area.
130. (HS 133) Cuboidcalcaneal facet outer shelf: 0 = absent; 1 = present.
131. (HS 134) Cuboidcalcaneal ventral facet: 0 = absent; 1 = present.
132. (HS 135) Spatial relationship between navicular and entocuneiform: 0 = entocuneiform anterior to navicular; 1 = entocuneiform extends proximally medial to the distal area of the navicular.
133. (HS 136) Navicular shelf between cuboid and astragalus: 0 = absent; 1 = present.
134. (HS 137) Navicular size*: 0 = navicular half to one third the size of the cuboid; 1 = navicular more than half the size of the cuboid to equal to it; 2 = navicular larger than the cuboid.
135. (HS 138) Mesocuneiform contact with navicular: 0 = absent; 1 = present.
136. (HS 139) Falcula on hallux: 0 = absent; 1 = present.
137. (HS 140) Prehallux: 0 = absent; 1 = present.
138. (HS 141) Mt IV proximal contact: 0 = ectocuneiform and cuboid; 1 = cuboid.
139. (modified HS 142) Mt V proximal process extends ventral to cuboid: 0=absent; 1=present.
140. (HS 143) Mt II and Mt III proximal ends*: 0 = Mt II extends more proximally than Mt III; 1 = equal; 2 = Mt III more proximal.
141. (HS 144) Hallux opposability (articulation Mt I with entocuneiform): 0 = not opposable; 1 = opposable.
142. (HS 145) Ridge on proximal articular facet of Mt I: 0 = absent; 1 = present.
143. (HS 146) Syndactyly or external fusion of digits II and III in the foot: 0 = absent, 1 = present.
144. (HS 147) Mt III thickness relative to that of Mt IV: 0 = Mt III thinner; 1 = Mt III and IV equal thickness or Mt IV thicker.
145. (HS 148) Mt III thickness relative to that of MtI*: 0 = Mt III thinner; 1 = Mt III and I equal; 2 = Mt III thicker than Mt I; 3 = Mt I absent.
146. (HS 149) Foot ungual phalanx of digit IV, proximal view*: 0 = larger dorsoventrally than mediolaterally; 1 = equal dimensions; 2 = larger mediolaterally than dorsoventrally.
147. (HS 150) Number of upper incisors*: 0 = five; 1 = four; 2 = three; 3 = two; 4 = one; 5 = none.
148. (HS 151) Number of lower incisors*: 0 = four; 1 = three; 2 = two; 3 = one; 4 = none.
149. (HS 152) Number of upper molars*: 0 = four; 1 = three; 2 = none.
150. (HS 153) Upper molar M2 shape: 0 = triangular or semi-triangular; 1 = rectangular or semi-square.
151. (HS 154) Paracone and metacone placement in M2: 0 = medial or buccal; 1 = buccal margin.
152. (HS 155) Paracone versus metacone size in M2*: 0 = pa > me; 1 = pa equals to me; 2 = pa < me.
153. (HS 156) Centrocrista shape: 0 = linear; 1 = V-shaped; 2 = absent.
154. (HS 157) Metaconule: 0 = absent or not-well developed; 1 = well-developed or enlarged.
155. (HS 158) Trigonid versus talonid width: 0 = trigonid wider than talonid; 1 = trigonid subequal to talonid or trigonid narrower than talonid.
Character refers to m2.
156. (HS 159) Paraconid on lower molars: 0 = absent; 1 = present.
157. (modified HS 160) Intersection of cristid obliqua with trigonid on m2*: 0: absent; 1: lingual to protocristid notch; 2 below protocristid notch; 3: labial to protocristid notch.
158. (HS 161) Upper incisor arcade shape*: 0 = U-shape; 1 = broad V-shape; 2 = long, narrow V-shape.
159. (HS 162) Upper dP1: 0 = absent; 1 = present to greatly reduced.
By analogy to recent marsupials with a similar dental formula and morphology, we assume that the first post-canine tooth in the upper series of Herpetotherium is dP1.
160. (HS 163) Lower dp2*: 0 = absent; 1 = greatly reduced; 2 = present.
161. (HS 164) Upper dP2*: 0 = absent; 1 = greatly reduced; 2 = present.
162. (HS 165) Upper incisors spatulate: 0 = no; 1 = yes.
163. (HS 166) Size upper I3/ vs. I2/*: 0 = I3/ > I2/; 1 = I3/ equal to I2/; 2 = I3/ < I2/.
164. (HS 167) Procumbent lower i3: 0 = no; 1 = yes.
165. (HS 168) Hypoconulid absent or present: 0 = absent; 1 = present.
166. (HS 169) Lower canine*: 0 = absent; 1 = greatly reduced; 2 = retained.
167. (HS 170) Upper canine: 0 = caniniform or premolariform; 1 = reduced or absent.
168. (HS 171) Number of roots on upper canine: 0 = two; 1 = one.
169. (HS 172) Lower i2 (i3 of Hershkovitz [13,18]) staggered or not staggered: 0 = not staggered; 1 = staggered.
170. (HS 173) Bunolophodonty or lophodonty developed: 0 = no; 1 = yes.
171. (HS 174) Selenodonty developed: 0 = no; 1 = yes.
172. (HS 175) Marsupial pattern of dental replacement: 0 = absent; 1 = present.
173. (HS 176) Parietal-alisphenoid or squamosal-frontal contact on braincase: 0 = parietal-alisphenoid; 1 = squamosal-frontal.
174. (HS 177) Width of frontals versus width of parietals: 0 = parietal wider or equal to frontal; 1 = parietal narrower than frontal. 
In the original definition of state “0” [1] noted as “(<” should have been “wider or equal”.
175. (HS 178) Angular process medially inflected: 0 = no; 1 = yes.
176. (HS 179) mandibular symphysis fused: 0 = no; 1 = yes.
177. (HS 180) Bones surrounding infraorbital canal in the orbit: 0 = maxilla + lacrimal; 1 = maxilla only.
178. (HS 181) Posterior-most point of premaxillo-nasal contact: 0 = anterior or at the canine; 1 = posterior to the canine.
179. (HS 182) Maximum maxilla (palatal portion) length/width ratio: 0 = ratio less or equal to 1.5; 1 = ratio larger than 1.5.
180. (HS 183) Maxillofrontal contact: 0 = absent; 1 = present.
181. (HS 184) Lacrimal tubercle: 0 = absent; 1 = present.
182. (HS 185) Alisphenoid tympanic wing*: 0 = absent; 1 = poorly developed; 2 = moderately developed; 3 = well-developed, extending to or near posterior lacerate foramen and paroccipital process.
183. (HS 186) Ectotympanic shape*: 0 = ring-shaped; 1 = moderately broadened; 2 = tubelike.
In Pucadelphys (MHNC 8266) the ectotympanic is ring-shaped.
184. (HS 187) Postglenoid process: 0 = absent; 1 = present.
185. (HS 188) Bony external auditory meatus separates ear canal from epitympanic recess: 0 = no; 1 = yes.
186. (HS 189) Fusion of ectotympanic with other bones of the skull: 0 = no; 1 = yes.
187. (HS 190) Postglenoid foramen: 0 = absent; 1 = present.
188. (HS 191) Postglenoid foramen position*: 0 = located posterior to postglenoid process; 1 = even with postglenoid process; 2 = anterior to postglenoid process and frequently encircled by squamosal.
189. (HS 192) Position of incisura tympanica: 0 = caudal or caudodorsal; 1 = located dorsally or anterior crus and posterior crus unite dorsally.
190. (HS 193) Size of incisura tympanica: 0 = narrow or absent; 1 = wide.
191. (HS 194) Foramen ovale position: 0 = lamina obturans / other bones; 1 = alisphenoid / petrosal; 2 = just alisphenoid; 3 = alisphenoid / squamosal.
192. (HS 195) Carotid foramen position: 0 = in basisphenoid; 1 = basisphenoid / basioccipital suture; 2 = basisphenoid / petrosal.
193. (HS 196) Transverse canal foramen: 0 = absent; 1 = anterior to carotid foramen; 2 = perforating pterygoid fossa; 3 = confluent with carotid foramen.
194. (HS 197) Intramural transverse canal: 0 = absent; 1 = present.
195. (HS 198) Dorsal margin of foramen magnum: 0 = formed by exoccipitals and supraoccipital; 1 = formed by exoccipitals.
196. (HS 199) Medial process of squamosal: 0 = absent; 1 = present.
197. (HS 200) Shape of nasals: 0 = posteriorly expanded; 1 = not posteriorly expanded.
198. (HS 201) Septomaxilla: 0 absent; 1 = present.
199. (HS 202) Palatal vacuities*: 0 = absent o just small foramina; 1 = present, restricted to palatine bones; 2 = present in both palatine and maxillary bones.
200. (HS 203) Premaxilla, palatal process: 0 = does not; 1 = does reach canine alveolus or it is immediately posterior to it.
201. (HS 204) Minor palatine foramen: 0 = absent; 1 = present.
202. (HS 205) Hypoglossal foramina* 0 = confluent with jugular foramen; 1 = one; 2 = two or more.
203. (HS 206) Optic foramen: 0 = absent; 1 = present.
This character is not observable in Mayulestes, contra Horovitz and Sánchez-Villagra [1].
204. (HS 207) Masseteric canal and dental canal in dentary: 0 = masseteric canal does not open into dental canal; 1 = masseteric canal opens into dental canal.
205. (HS 208) Malleolar neck: 0 = long relative to head and manubrium; 1 = short.
206. (HS 209) Ossicular axis*: 0 = > 20 degrees; 1 = 10 to 20 degrees; 2 = < 10 degrees.
207. (HS 210) Manubrial-incudal lever-arm ratio: 0 = equal or smaller than 1.6; 1 = larger than 1.6.
208. (HS 211) Stapedial ratio: 0 = < 1.8; 1 = > 1.8.
209. (HS 212) Stapedial foramen visible: 0 = absent; 1 = present.
210. (HS 213) Bullate stapes: 0 = not bullate; 1 = bullate.
211. (HS 223) Paraseptal cartilage dips down vertically by the side of nasopalatine duct: 0 = no; 1= yes.
212. (HS 224) Paraseptal cartilage shape: 0 = outer bar connects with uppermost portion of paraseptal cartilage; 1 = with middle portion, dorsal process short; 2 = with middle portion, dorsal process long; 3 = outer bar fused to paraseptal cartilage instead of being an isolated structure.
213. (HS 225) Portion of paraseptal cartilage is ring-shaped in cross section: 0 = no; 1 = yes.
214. (HS 226) Sperm pairing in epididymis: 0 = no; 1 = yes.
215. (HS 227) Pouch type in mammary area: 0 = type 1; 1 = type 5; 2 = type 6; 3 = no marsupium or skin folds develop during the reproductive period; 4 = thin marsupium-like structure develops during reproductive period.
216. (HS 228) Mammary count *: 0 = 0 teats; 1 = 2 teats; 2 = 4 teats; 3 = 5-8 teats; 4 = 9 teats or more.
217. (HS 229) Caecum: 0 = absent; 1 = present.
218. (HS 230) Fasciculus aberrans in brain: 0 = absent; 1 = present.
219. (LA1) Complete wall separating cavum supracochleare from cavum epiptericum: 0 = absent; 1 = present.
References: Wible ([19], ch. 2), Wible and Hopson ([20], ch. 6), Rougier et al. ([21,22], ch. 40), Rougier et al. ([23], ch. 128), Wible, et al. ([24], ch. 128), Luo et al. ([25], ch. 203), Sánchez-Villagra and Wible ([26], ch. 11).
220. (LA2) Cavum epiptericum floored by: 0 = petrosal; 1 = petrosal and alisphenoid; 2 = primarily or exclusively by alisphenoid; 3 = primarily open as piriform fenestra.
References: ([20], ch. 4), Rougier et al. ([21], ch. 35), Rougier et al. ([23], ch. 109), Wible et al. ([24], ch. 109).
In Andinodelphys, Pucadelphys and Mayulestes, the trigeminal ganglion is posteriorly floored by the anterior lamina of the petrosal. The contribution of the petrosal to the floor of the cavum epiptericum being substantial, those taxa are coded “1”. The platypus has a cavum epiptericum partially floored by the lamina obturans and appositional bone from the petrosal [20]. The echidna has a complete floor formed by the lamina obturans, appositional bone from the petrosal, ectopterygoid, and palatine [27]. Though those morphologies do not correspond exactly to any of the scores, both genera of monotremes were scored 0, as the petrosal is part of the floor of the cavum epiptericum.
221. (LA3) Fossa subarcuata: 0 = smaller than its aperture (i.e., conical shape); 1 = larger than its aperture (i.e., spherical shape).
		Reference: Ladevèze ([11], ch. 2).
222. (LA4) Posterior exposure of the pars mastoidea: 0 = dorsoventrally elongated and approximately flat; 1 = rounded and bulbous due to the excavation of the fossa subarcuata.
		Reference: Ladevèze ([11], ch. 3).
223. (LA5) Expansion of the crista petrosa that forms a thin lamina covering the anterolateral part of the fossa subarcuata: 0 = absent; 1 = present.
		Reference: Ladevèze and Muizon ([12], ch. 122).
224. (LA6) Anterior lamina of petrosal exposure on the lateral wall of the braincase: 0 = present and large; 1 = rudimentary; 2 = absent.
		References: Wible [19], Wible and Hopson ([20], ch. 1, 2), Rougier et al. ([23], ch. 108), Wible et al. ([24], ch. 108).
225. (LA7) Anterior lamina of petrosal: 0 = shows a large depression that may have received a part of temporal lobe of the brain and the posterior part of the trigeminal ganglion; 1 = shows no depression.
		References: Ladevèze ([11], ch. 6).
226. (LA8) Internal acoustic meatus and fossa subarcuata: 0 = subequal and separated by a sharp wall; 1 = internal acoustic meatus narrower than the opening of the fossa subarcuata and separated from the latter by a thick shelf of bone.
		Reference: Ladevèze and Muizon ([12], ch. 125).
		Tachyglossus is scored "?" as the conformation is peculiar: the fossa subarcuata forms a shallow but large fossa.
227. (LA9) Internal acoustic meatus: 0 = deep with thick prefacial commissure; 1 = shallow with thin prefacial commissure.
		References: Rougier et al. ([23], ch. 153), Wible et al. ([24], ch.153).
228. (LA10) Deep groove for internal carotid artery excavated on anterior pole of promontorium: 0 = absent; 1 = present.
		References: Muizon et al. [28], Rougier et al. ([23], ch. 148), Wible et al. ([24], ch. 148), Sánchez-Villagra and Wible ([26], ch. 16), Horovitz and Sánchez-Villagra ([1], ch. 220).
229. (LA11) Deep and large fossa for the tensor tympani muscle excavated on the anterolateral aspect of promontorium, creating a battered ventral surface of the promontorium: 0 = absent; 1 = present.
References: Wible ([19], ch. 12).
230. (LA12) Epitympanic wing of petrosal: 0 = absent; 1 = present.
		References: Rougier et al. ([23], ch. 122); Wible et al. ([24], ch. 122).
This term is used for outgrowths of any basicranial bones that contribute to the tympanic roof [29]. An epitympanic wing extends medially from the promontorium in many therians ([21], 1998).
231. (modified LA13) Epitympanic wing of petrosal: 0 = flat (0), or 1 = confluent with bulla.
		References: Rougier et al. ([23], ch. 122); Wible et al. ([24], ch. 122).
232. (LA14) Lateral flange: 0 = large and lateral to promontorium; 1 = greatly reduced or absent.
References: Wible ([19], ch. 3), Rougier et al. ([21], ch. 29), Rougier et al. ([23], ch. 126), Wible et al. ([24], ch. 126).
In non-therian mammals, a large shelf of bone extends lateral to and on the whole length of the promontorium. It is the lateral trough, the lateral edge of which is downturned to form the lateral flange [30]. In therians, the lateral flange is either greatly reduced or absent [30], while a lateral trough persists in Andinodelphys, Pucadelphys and Mayulestes (pers. obs.).
233. (LA15) Broad shelf of bone surrounding fenestra cochleae and making a separation between it and aqueductus cochleae: 0 = absent; 1 = present.
		Reference: Ladevèze and Muizon ([12], ch. 132).
Such a structure was observed on the petrosals of Pucadelphys and Andinodelphys. In monotremes, the fenestra cochleae is not separated from the jugular foramen.
234. (LA16) Rostral tympanic process of petrosal: 0 = absent; 1 = present as a distinct crest or erected process.
		References: Wible ([19], ch. 9), Rougier et al. ([23], ch. 130), Wible et al. ([24], ch. 130).
		The presence of a low and tiny ridge or tubercle, anterolateral to the fenestra vestibuli is not regarded as equivalent to the development of a tympanic process, since it is not a process, i.e., a raised shelf of bone. Pediomys and Didelphodon are thus coded “0”. The monotreme Tachyglossus exhibits a large and broad process on the ventral surface of the promontorium. Nevertheless, the ontogenies of the promontorium processes in monotremes and metatherians are not entirely comparable. The process observed in monotremes results from an co-ossification of Reichert's cartilage and the cochlear capsule [31,32], whereas that of metatherians results from a periosteal outgrowth from the ossifying cochlear capsule [33]. Thus, Tachyglossus is here scored as lacking a rostral tympanic process of petrosal.
235. (LA17) Rostral tympanic process of petrosal: 0 = forms an anterolaterally directed wing, sometimes contacting the ectotympanic, that does not extend on the whole length of the promontorium; 1 = that does extend on the whole length of the promontorium.
References: Sánchez-Villagra and Wible ([26], ch. 7), Horovitz and Sánchez-Villagra ([1], ch. 215).
236. (LA18) Tympanic aperture of hiatus Fallopii*: 0 = dorsal (0); 1 = intermediate; 2 = ventral.
Reference: Sánchez-Villagra and Wible ([26], ch. 12), Horovitz and Sánchez-Villagra ([1], ch. 218).
237. (LA19) Stylomastoid foramen: 0 = absent; 1 = present.
References: Archer [34], Wroe et al. ([35], ch. 54).
The monotreme Tachyglossus has an open facial sulcus, which runs into a stylomastoid foramen. This conformation is different from that defined by Archer [34] and Wroe, et al. [35], i.e., a facial canal that opens onto a stylomastoid foramen within petrosal.
238. (LA20) Inferior petrosal sinus: 0 = intrapetrosal; 1 = between petrosal, basisphenoid and basioccipital; 2 = endocranial.
		References: Rougier et al. ([21,22], ch. 42), Rougier et al. ([23], ch. 151), Wible et al. ([24], ch. 151).
239. (LA21) Mastoid exposure: 0 = large, 1 = narrow; 2 = reduced pars mastoidea, internal to the braincase and wedged between the squamosal and exoccipital.
Reference: Ladevèze ([11], ch. 18).
240. (LA22) Mastoid tympanic process: 0 = large and vertical (0), 1 = small, slanted, and nodelike, on the posterolateral border of the stylomastoid notch and continuous with squamosal; 2 = indistinct to absent.
References: Wible ([19], ch. 7), Rougier, et al. ([23], ch. 131), Wroe, et al. ([35], ch. 67), Wible, et al. ([24], ch. 131), Ladevèze (2004, ch. 19).
Two scores differ from Wible, et al. [24]: Pucadelphys and Andinodelphys are scored as having a slanted mastoid tympanic process, quite well-developped and nodelike, that form a posterolateral wall for the stylomastoid notch and that follows the squamosal.
241. (LA23) Caudal tympanic process of petrosal: 0 = absent; 1 = present.
References: Wible ([19], ch. 8), Wible and Hopson ([20], ch. 26), Rougier et al. ([21], ch. 18), Rougier et al. ([23], ch. 132), Wible et al. ([24], ch. 132), Luo et al. ([25], ch. 200).
According to MacPhee [29], the term ‘‘caudal tympanic process of petrosal’’ is applied to the process that arises from the tympanic surface of the pars canalicularis in the area posterior to cochlear fossula and medial to the stylomastoid opening.
242. (LA24) Caudal tympanic process of petrosal: 0 = forms a small crest that does not wholly floor the postpromontorial sinus; 1 = forms an expanded lamina that floors the postpromontorial sinus.
Reference: Ladevèze ([11], ch. 20).
243. (LA25) Petrosal plate: 0 = absent; 1 = present.
References: Wible ([19], ch. 8), Sánchez-Villagra and Wible ([26], ch. 8), Horovitz and Sánchez-Villagra ([1], ch. 216), Ladevèze ([11], ch. 21).
The petrosal plate (sensu [36]) is formed by the joined caudal and rostral tympanic processes of the petrosal. This feature is linked to a pneumatisation of the petrosal (see Dromiciops and dasyurids) and is also found in the didelphid Caluromys and in the fossil microbiotheriid Microbiotherium [37].
244. (LA26) Fossa incudis and epitympanic recess: 0 = continuous; 1 = separated by a distinct ridge.
References: Rougier et al. ([23], ch. 137), Wible et al. ([24], ch. 137).
An epitympanic recess is found in multituberculates, Vincelestes, and therians, whereas a fossa incudis is more widely distributed among mammaliaforms [21]. Some metatherians have those two fossa separated by a distinct ridge (i.e., Didelphodon, Pediomys, borhyaenids, and some dasyurids [23]).
245. (LA27) Petrosal crest: 0 = absent; 1 = present.
References: Archer [34], Ladevèze ([11], ch. 23).
Archer [34] defined the petrosal crest as the bony edge which separates the anterior part of the epitympanic recess from the posterior part of the hypotympanic sinus of the alisphenoid.
246. (LA28) Petrosal contribution to the lateral wall of the epitympanic recess: 0 = absent; 1 = present.
Reference: Ladevèze ([11], ch. 24).
In extant metatherians, the epitympanic recess is laterally bordered by the squamosal, which supports the external acoustic meatus. On isolated petrosals, a shelf of bone of the petrosal bounds laterally the epitympanic recess, but is covered by the squamosal in entire skulls. In Andinodelphys, Pucadelphys and Mayulestes, the epitympanic recess is divided and lies in both squamosal and petrosal.
247. (LA29) Petrosal contribution to the lateral wall of the epitympanic recess: 0 = massive, large shelf of bone, sometimes rounded (0), 1 = slander and triangular; 1 = forming a thin lamina.
Reference: Ladevèze ([11], ch. 24).
248. (LA30) Prootic canal: 0 = present; 1 = absent.
References: Wible ([19], ch. 17), Wible and Hopson ([20], ch. 18), Wible and Hopson [38], Wroe et al. ([35], ch. 77), Sánchez-Villagra and Wible ([26], ch. 9, 10).
249. (LA32) Imprint of the transverse sinus bifurcation on the petrosal: 0 = absent; 1 = present.
Reference: Ladevèze ([11], ch. 27).
This character can be coded only on isolated petrosals.
250. (LA33) Foramina on the sigmoid sinus and/or prootic sinus, apparently connecting both vessels (i.e., sigmoid sinus vein): 0 = absent; 1 = present.
		Reference: Ladevèze and Muizon ([12], ch. 150).
251. (LA34) Vascular groove medially adjacent to prootic sinus sulcus, on the pars mastoidea (i.e., prootic sinus vein or connection): 0 = absent; 1 = present.
		Reference: Ladevèze and Muizon ([12], ch. 151).
252. (LA35) Posttemporal sulcus on the squamosal surface of the petrosal: 0 = present; 1 = absent.
References: Wible and Hopson [38], Rougier et al. ([23], ch. 144), Wible et al. ([24], ch. 144), Sánchez-Villagra and Wible ([26], ch. 13), Horovitz and Sánchez-Villagra ([1], ch. 219).
253. (LA36) Posttemporal notch/foramen: 0 = present; 1 = absent.
References: Wible ([19], ch. 24), Rougier et al. ([23], ch. 144), Wible et al. ([24], ch. 144), Luo et al. ([25], ch. 251), Sánchez-Villagra and Wible ([26], ch. 14).
254. (LA37) Transpromontorial sulcus: 0 = present; 1 = absent.
References: Wible [39], Rougier et al. ((23], ch. 146), Wible et al. ([24], ch. 146), Luo, et al. ([25], ch. 220).
255. (LA38) Sulcus for stapedial artery: 0 = present; 1 = absent.
References: Wible [40], Wible [[19], ch. 16), Rougier et al. ([23], ch. 147), Wible et al. ([24], ch. 147), Luo et al. ([25], ch. 219), Horovitz and Sánchez-Villagra ([1], ch. 221).
256. (LA39) Cochlear coiling: 0 = absent or less than 300°; 1 = fully coiled (more than 360°).
References: Rougier et al. ([23], ch. 129), Wible et al. ([24], ch. 129), Luo et al. ([25], ch. 194), Horovitz and Sánchez-Villagra ([1], ch. 222).
257. (LA45) Tympanic sinus formed in the lateral trough (or anterolateral expansion of the pars canalicularis): 0 = absent; 1 = present.
		Reference: Ladevèze and Muizon ([12], ch. 162).
A large fossa excavated on the "lateral trough", or on the lateral expansion of the pars canalicularis was observed on the petrosals of the Tiupampan taxa Mayulestes, Pucadelphys, and Andinodelphys, and the Itaboraían Type II, and was interpreted as a tympanic sinus. This deep sinus is a large subtriangular fossa that lies anterolateral to the fossa for the tensor tympani muscle and anterior to the epitympanic recess and petrosal crest. In Mayulestes, it corresponds to the posterior half of what Muizon [41] named “alisphenoid hypotympanic sinus” even if the term “hypotympanic” is not appropriated in this case. In Mayulestes this sinus is completed by the alisphenoid and squamosal and approximates the condition of extant metatherians, while in Pucadelphys and Andinodelphys it is completed by the posterior edge of the squamosal (medial process). In the borhyaenids Notogale and Sallacyon such a sinus is found on the lateral trough, and is completed by the alisphenoid in a hypotympanic sinus.
258. (LA47) (Hypo)-tympanic sinus formed by petrosal and squamosal: 0 = absent; 1 = present.
		Reference: Ladevèze and Muizon ([12], ch. 164).
259. (LA48) (Hypo)-tympanic sinus formed by alisphenoid and petrosal, or by alisphenoid only: 0 = absent; 1 = present.
		References: Rougier et al. ([23], ch. 140), Wroe et al. ([35], ch. 56), Wible et al. ([24], ch. 140), Luo et al. ([25], ch. 223), Luo et al. ([14], ch. 312), Ladevèze and Muizon ([12], ch. 165).
260. Labial posterior cingulum in lower molars: 0 = absent; 1 = present.
References: Rougier et al. [42], Cifelli and Muizon [43], Clemens and Lillegraven ([9], p. 64), Voss and Jansa [44].

Phylogenetic analysis: The data matrix (Table S2) was submitted to a maximum parsimony analysis [45] using paup* 4.0b10 for unix [46]. polymorphic terminals (referred to as “multistate taxa” by swofford [46]) were considered “heterogeneous”, so the command “pset mstaxa=polymorph” was used. ornithorhynchus was the designated as root of the tree (indicated as “outgroup” by paup). we conducted 1,000 replicates of a heuristic search with stepwise random addition sequence of taxa, and tree bisection-reconnection. the resulting tree had length = 1013, ci (consistency index) = 0.43, ri (retention index) = 0.65, rc (rescaled consistency index) = 0.28 (Figure S6). A list of unambiguous synapomorphies is presented in Table S3, following the node numbering indicated in Figure S6.
Three kinds of support measure were calculated: Goodman-Bremer support [47-50], bootstrap and jackknife. Goodman-Bremer support was calculated by searching trees of increasing length, one step at a time, and computing a consensus tree of results obtained with each additional step (Figure S6).
We also conducted bootstrap and jackknife searches to estimate degree of branch support [51-53]. We did not interpret degree of support from a statistical point of view but as a way of understanding amount of support of different branches relative to each other. Bootstrap was computed with 50% deletion of characters, 1,000 bootstrap replicates and 30 heuristic search replicates (with stepwise random addition sequence of taxa and tree bisection-reconnetion) for each bootstrap replicate.
According to computations by Harshman ([54]: table 3), the size of a data set affects the bootstrap support that a node will receive if other variables are kept proportional to overall size of the data set. For example, let us suppose we have two data sets for the same set of taxa, one consisting of 10 characters and the other of 50. The number of characters (r) supporting a certain node AB and the number of characters supporting mutually exclusive alternative solutions BC and AC (s and t) also show a proportion of 5. For example, r,s,t are equal to 2,1,1 respectively in the 10-character data set and 10,5,5 in the 50 character data set. The expected bootstrap support for node AB is equal to 48% (and the node is therefore not be recovered) in the 10-character data set whereas it would be of 81% in the 50-character data set. Morphological data sets are commonly smaller in number of characters than most DNA sequence data sets in proportion to their number of taxa. In addition, when fossil taxa are included, many characters display missing entries. We therefore concluded that deleting half of the characters at a time (a setting that was designed for DNA sequence data sets) does not leave much information to work with in our data set and may therefore not be the most adequate way of estimating support for our tree. Many recovered branches received support below 50% in the bootstrap tree (and were therefore not collapsed) even though the amount and quality of support for many of those branches varied. In other words the method did not allow discrimination of degrees of support for many portions of the tree and it was therefore not very useful to make too many internal comparisons of support among branches within the tree.
We also conducted another set of trials in which instead of using bootstrap, we used jackknife searches which sample characters only once and in this way we avoided the rather unnecessary weighting of characters that results from resampling them when one uses bootstrap searches. In addition, we adjusted the magnitude of character deletion to be able to discriminate degree of support throughout our tree. We performed deletions of 25% of characters at a time. With this readjustment, of the 11 branches that had received zero support in the bootstrap search, two of them still received no support and the remaining 9 received support ranging between 73 and 60. Therefore we deemed this adjustement adequate enough to be able to discriminate better different degrees of relative support among branches for our tree (Figure S6).

		References
1.	Horovitz I, Sánchez-Villagra MR (2003) A morphological analysis of marsupial mammal higher-level phylogenetic relationships. Cladistics 19:181-212.
2.	Sánchez-Villagra MR, Ladevèze S, Horovitz I, Argot C, Hooker JJ, et al. (2007) Exceptionally preserved North American Paleogene metatherians: adaptations and discovery of a major gap in the opossum fossil record. Biol Lett 3:318-322.
3.	Horovitz I, Ladeveze S, Argot C, Macrini TE, Martin T, et al. (2008) The anatomy of Herpetotherium cf. fugax COPE, 1873, a metatherian from the Oligocene of North America. Palaeontogr Abt A 284:109-141.
4.	Bown TM, Rose KD (1979) Mimoperadectes, a new marsupial, and Worlandia, a new dermopteran, from the lower part of the Willwood Formation (Early Eocene), Bighorn Basin, Wyoming. Contrib Mus Paleontol Univ Michigan 25:89-104.
5.	Strait SG (2001) New Wa-0 mammalian fauna from Castle Gardens in the southeastern Bighorn Basin. Univ Mich Pap Paleontol 33:127-143.
6.	Gingerich PD, Smith T (2006) Paleocene-Eocene land mammals from three new latest Clarkforkian and earliest Wasatchian wash sites at Polecat Bench in the northern Bighorn Basin, Wyoming. Contrib Mus Paleontol Univ Michigan 31:245-303.
7.	Clemens WA (1966) Fossil mammals of the type Lance Formation, Wyoming. Part II. Marsupialia. Univ Calif Publ Geol Sci 62:1-122.
8.	Crochet JY (1980) Les marsupiaux du Tertiaire d'Europe. Paris: Editions de la Fondation Singer-Polignac. 279 p.
9.	Clemens WA, Lillegraven JA (1986) New Late Cretaceous, North American advanced therian mammals that fit neither the marsupial or eutherian molds. Univ Wyo Contrib Geol Spec Pap 3:55-85.
10.	Fox RC (1983) Notes on the North-American Tertiary marsupials Herpetotherium and Peradectes. Can J Earth Sci 20:1565-1578.
11.	Ladevèze S (2004) Metatherian petrosals from the Late Paleocene of Itaboraí (Brazil), and their phylogenetic implications. J Vertebr Paleontol 24:202-213.
12.	Ladevèze S, Muizon C de (2007) The auditory region of early Paleocene Pucadelphydae (Mammalia, Metatheria) from Tiupampa, Bolivia, with phylogenetic implications. Palaeontology 50:1123-1154.
13.	Hershkovitz P (1995) The staggered marsupial third lower incisor: hallmark of Cohort Didelphimorphia, and description of a new genus and species with staggered i3 from the Albian (Lower Cretaceous) of Texas. Bonn Zool Beitr 45:153-169.
14.	Luo Z-X, Ji Q, Wible JR, Yuan CX (2003) An Early Cretaceous tribosphenic mammal and metatherian evolution. Science 302:1934-1940.
15.	Szalay F, Trofimov B (1996) The Mongolian Late Cretaceous Asiatherium, and the early phylogeny and paleobiogeography of Metatheria. J Vertebr Paleontol 16:474-509.
16.	Szalay FS (1994) Evolutionary history of the marsupials and an analysis of osteological characters. Cambridge: Cambridge University Press. 481 p.
17.	Horovitz I, Sánchez-Villagra MR (2003) A morphological analysis of marsupial mammal higher-level phylogenetic relationships. Cladistics 19:181-212.
18.	Hershkovitz P (1999) Dromiciops gliroides Thomas, 1894, last of the Microbiotheria (Marsupialia), with a review of the family Microbiotheriidae. Fieldiana Zool New Ser 93:1-60.
19.	Wible JR (1990) Petrosals of Late Cretaceous marsupials from North America, and a cladistic analysis of the petrosal in therian mammals. J Vertebr Paleontol 10:183-205.
20.	Wible JR, Hopson JA (1993) Basicranial evidence for early mammal phylogeny. In: Szalay FS, Novacek MJ, McKenna MC, editors. Mammal Phylogeny: Mesozoic differentiation, multituberculates, monotremes, early therians, and marsupials. New York: Springer-Verlag. Vol. 1. pp. 45-62.
21.	Rougier GW, Wible JR, Hopson JA (1996) Basicranial anatomy of Priacodon fruitaensis (Triconodontidae, Mammalia) from the late Jurassic of colorado, and a reappraisal of mammaliaform interrelationships. Am Mus Novit 3183:1-38.
22.	Rougier GW, Wible JR, Novacek MJ (1996) Middle-ear ossicles of the multitberculate Kryptobaatar from the Mongolian Late Cretaceous: Implications for Mammaliamorph relationships and the evolution of the auditory apparatus. Am Mus Novit 3187:1-43.
23.	Rougier GW, Wible JR, Novacek MJ (1998) Implications of Deltatheridium specimens for early marsupial history. Nature 396:459-463.
24.	Wible JR, Rougier GW, Novacek MJ, McKenna MC (2001) Earliest eutherian ear region: a petrosal referred to Prokennalestes from the Early Cretaceous of Mongolia. Am Mus Novit 3322:1-44.
25.	Luo Z-X, Kielan-Jaworowska Z, Cifelli RL (2002) In quest for a phylogeny of Mesozoic mammals. Acta Palaeontol Pol 47:1-78.
26.	Sánchez-Villagra MR, Wible JR (2002) Patterns of evolutionary transformation in the petrosal bone and some basicranial features in marsupial mammals, with special reference to didelphids. J Zool Syst Evol Res 40:26-45.
27.	Kuhn HJ, Zeller U (1987) The cavum epiptericum in monotremes and therian mammals. In: Kuhn HJ, Zeller U, editors. Morphogenesis of the mammalian skull. Hamburg: Verlag Paul Varey. pp. 51-70.
28.	Muizon C de, Cifelli RL, Paz RC (1997) The origin of the dog-like borhyaenoid marsupials of South America. Nature 389:486-489.
29.	MacPhee RDE (1981) Auditory regions of primates and eutherian insectivores: morphology, ontogeny, and character analysis. Contrib Primatol 18:1-282.
30.	Wible JR, Rougier GW, Novacek MJ, McKenna MC, Dashzeveg D (1995) A mammalian petrosal from the early Cretaceous of Mongolia: Implications for the evolution of the ear region and mammaliamorph interrelationships. Am Mus Novit 3149:1-19.
31.	Kuhn HJ (1971) Die Entwicklung und Morphologie des Schädels von Tachyglossus aculeatus. Abh Senckenberg Naturforsch Ges 528:1-224.
32.	Presley R (1980) The braincase of Recent and Mesozoic therapsids. Mém Soc géol France n s 139:159-162.
33.	Maier W (1987) Der Processus angularis bei Monodelphis domestica (Didelphidae; Marsupialia) und seine Beziehungen zum Mittelohr: Eine ontogenetische und evolutionsmorphologische Untersuchung. Gegenbaurs Morphol Jahrb 133:123-161.
34.	Archer M (1976) The basicranial region of marsupicarnivores (Marsupialia), interrelationships of carnivorous mammals, and affinities of the insectivorous marsupial peramelids. Zool J Linn Soc 59:217-322.
35.	Wroe S, Ebach M, Ahyong S, Muizon C de, Muirhead J (2000) Cladistic analysis of dasyuromorphian (Marsupialia) phylogeny using cranial and dental characters. J Mammal 81:1008-1024.
36.	Van der Klaaw C (1931) The auditory bulla in some fossil mammals, with a general introduction to this region of the skull. Bull Am Mus Nat Hist 62:1-352.
37.	Marshall LG (1982) Systematics of the South American marsupial Family Microbiotheriidae. Fieldiana Geol New Ser 10:1-75.
38.	Wible JR, Hopson JA (1995) Homologies of the prootic canal in mammals and non-mammalian cynodonts. J Vertebr Paleontol 15:331-356.
39.	Wible JR (1986) Transformations in the extracranial course of the internal carotid artery in mammalian phylogeny. J Vertebr Paleontol 6:313-325.
40.	Wible JR (1987) The eutherian stapedial artery: character analysis and implications for supraordinal relationships. Zool J Linn Soc 91:107-135.
41.	Muizon C de (1998) Mayulestes ferox, a borhyaenoid (Metatheria, Mammalia) from the early Paleocene of Bolivia.  Phylogenetic and palaeobiologic implications. Geodiversitas 20:19-142.
42.	Rougier GW, Wible JR, Novacek MJ (2004) New specimen of Deltatheroides cretacicus (Metatheria, Deltatheroida) from the Late Cretaceous of Mongolia. Bull Carnegie Mus Nat Hist 36:245-266.
43.	Cifelli RL, Muizon C de (1997) Dentition and jaw of Kokopellia juddi, a primitive marsupial or near-marsupial from the Medial Cretaceous of Utah. J Mamm Evol 4:241-258.
44.	Voss RS, Jansa SA (in press) Phylogenetic relationships and classification of didelphid marsupials, an extant radiation of New World metatherian mammals. Bull Am Mus Nat Hist.
45.	Farris JS (1970) Methods for computing Wagner trees. Syst Zool 19:83-92.
46.	Swofford D (2002) PAUP* 4.0b10. Program and Documentation. Sunderland, Massachussets: Sinauer Associates. 
47.	Bremer K (1988) The limits of amino-acid sequence data in angiosperm phylogenetic reconstruction. Evolution 42:795-803.
48.	Bremer K (1994) Branch support and tree stability. Cladistics 10:295-304.
49.	Goodman M, Olson CB, Beeber JE, Czelusniak J (1982) New perspectives in the molecular biological analysis of mammalian phylogeny. Acta Zool Fenn 169:19-35.
50.	Grant T, Kluge AG (2008) Credit where credit is due: The Goodman-Bremer support metric. Mol Phylogenet Evol 49:405-406.
51.	Farris JS, Albert VA, Källersjö M, Lipscomb D, Kluge AG (1996) Parsimony jackknifing outperforms neighbor-joining. Cladistics 12:99-124.
52.	Felsenstein J (1985) Confidence limits on phylogenies: an approach using the bootstrap. Evolution 39:783-791.
53.	Felsenstein J (2004) Inferring phylogenies. Sunderland, Massachuseetts: Sinauer Associates, Inc. 664 p.
54.	Harshman J (1994) The effect of irrelevant characters on bootstrap values. Syst Biol 43:419-424.
