# Supplementary material for: Cranial Anatomy of the Earliest Marsupials and the Origin of Opossums
Source: PLoS One. 2009 Dec 16;4(12):e8278. doi: 10.1371/journal.pone.0008278 (PMC2789412; doi:10.1371/journal.pone.0008278)
Supplement: Text S3 — Cranial and dental measurements and notes about Table S1 (0.03 MB DOC) [file pone.0008278.s003.doc]

**Cranial and dental measurements and notes about Table S1**

**Measurements for *Mimoperadectes houdei* USNM 482355**:

Dental measurements are presented in Table S1.

Cranial measurements (key in Figure S4):

A = 24.7

Approximate distance from posterior concavity of supraorbital process to posterior end of sagittal crest (although the crest may be missing a small portion).

B = 7.62

Minimal width of postorbital constriction.

C = 10.85

Width of skull from middle of sagittal crest to concavity above external auditory meatus.

D = 22.0

Alveolar length of toothrow (canine included): distance from anterior edge of alveolus for canine to posterior edge of alveolus for M4.

E = 18.81

Alveolar length of toothrow (canine excluded).

F = 10.24

Alveolar length of molar toothrow.

**Measurements for *Mimoperadectes labrus:***

Upper dentition only (for comparison), presented in Table S1, from Gingerich ([1]:22).

# Reference

1. Gingerich PD (1989) New earliest Wasatchian mammalian fauna from the Eocene of northwestern Wyoming: composition and diversity in a rarely sampled high-floodplain assemblage. Univ Mich Pap Paleontol 28:1-97.
