# Supplementary material for: Cranial Anatomy of the Earliest Marsupials and the Origin of Opossums
Source: PLoS One. 2009 Dec 16;4(12):e8278. doi: 10.1371/journal.pone.0008278 (PMC2789412; doi:10.1371/journal.pone.0008278)
Supplement: Table S1 — Dental measurements. W = width, L = length, ratio = W/L, P = upper premolar, M = upper molar, left teeth except for M4. Measurements in milimeters taken with a Zeiss Discovery V12 Stereomicroscope with an Axiocam digital Camera and Axiovision software. See Text S3 for source of Mimoperadectes labrus measurements (0.03 MB DOC) [file pone.0008278.s004.doc]

| Upper dentition | *Mimoperadectes* *houdei*  USNM 482355 (holotype) | *Mimoperadectes labrus*  UM 66144 (holotype) |
| --- | --- | --- |
| Canine L | 3.40 |  |
| Canine W | 1.98 |  |
| P1 L | 1.23 |  |
| P1 W | 0.67 |  |
| P2 L | 2.75 |  |
| P2 W | 1.50 |  |
| M1 L | 3.28 | 3.1 |
| M1 W | 3.47 | 3.0 |
| **M1 surface** | **11.38** | **9.3** |
| M2 L | 2.96 | 3.2 |
| M2 W | 3.77 | 3.6 |
| **M2 surface** | **11.16** | **11.52** |
| M1 surface / M2 surface | **1.02** | **0.81** |
| M3 L | 3.10 | 3.1 |
| M3 W | 4.2 | 4.0 |
| **M3 surface** | **13.02** | **12.4** |
| M4 L | 2.49 |  |
| M4 W | 3.26 |  |
| **M4 surface** | **8.11** |  |
