# Supplementary material for: Cranial Anatomy of the Earliest Marsupials and the Origin of Opossums
Source: PLoS One. 2009 Dec 16;4(12):e8278. doi: 10.1371/journal.pone.0008278 (PMC2789412; doi:10.1371/journal.pone.0008278)
Supplement: Table S2 — Data matrix. Polymorphic entries, a = (01), b = (02), c = (12), d = (012). Inapplicable entries are represented with “- ” and unknown as “?”. Mimo/peradectes stands for Mimoperadectes-Peradectes (0.29 MB DOC) [file pone.0008278.s005.doc]

|  | 1 | 11 | 21 | 31 | 41 | 51 |
| --- | --- | --- | --- | --- | --- | --- |
|  | | | | | | | | | | | | |
| *Ornithorhynchus* | 001111?100 | 2101110112 | 000?001021 | 00100101-- | -101101010 | –111010010 |
| *Tachyglossus* | 101011?100 | 2000110020 | 000?001021 | 00100101-- | -100101010 | –111010011 |
| *Vincelestes* | 10010???11 | 2?0011?112 | 1010?2?101 | ????10??12 | 1100111001 | 11101111?? |
| *Ukhaatherium* | 0?0?1????? | ?????????? | 1?101?0?0? | ????????0? | 210?1??001 | ?100?1?1?0 |
| *Asioryctes* | 000?011010 | 1?000-?100 | 1011?????? | ?????????? | ?????????? | ?????????? |
| *Deltatheridium* | ?????????? | ?????????? | ?????????? | ?????????? | ?????????? | ?????????? |
| *Mayulestes* | 00010??011 | 0?00?????? | ?01???01?? | ????10000? | 1111111011 | 0010111111 |
| *Pucadelphys* | 00010??011 | 0?011??110 | 10111?0??1 | 0???20000? | 2011111001 | 01101111?1 |
| *Andinodelphys* | 00010??011 | ??0????01? | 1?11??0??1 | ????2?000? | 2??1111001 | 1??01111?1 |
| *Didelphis* | 1aa101?10a | 1000100122 | 1011a20001 | 0101200011 | 10011110a1 | aa10111100 |
| *Monodelphis* | a10101?110 | 1000100110 | 1011120001 | 0101200002 | 2a101c1011 | 0110011100 |
| *Caenolestes* | 000001?110 | ?1001101ad | 1110?2??01 | 0001200002 | 0021101001 | 0110111100 |
| *Dasyuroides* | aa0001?110 | 0a00101100 | 1a110?0101 | 0001200002 | 2020111001 | 0010011100 |
| *Dasyurus* | 11a101?110 | 1a0010?1ca | 1a11a20101 | 00012000?? | ?a210111a1 | a000011100 |
| *Phascogale* | 11?001?110 | 11001011ad | 1111020101 | 0001200002 | 1021121001 | 0000011100 |
| *Notoryctes* | 00a011?1?0 | a?20?????0 | 1?10010021 | 0000300002 | 2001010101 | 1110010100 |
| *Echymipera* | 0001011110 | 010011112d | 1110120121 | 1001311002 | 1111130011 | 0000011100 |
| *Perameles* | 00000111?0 | 010011012d | 1?10120121 | 1001311002 | 0111120001 | 0000011100 |
| *Dromiciops* | a0000101?0 | 000010111c | 1?11010001 | 0101100012 | 2100111001 | 1010001100 |
| *Trichosurus* | 1a0101?110 | 0000101112 | 11110100c1 | 0101100011 | 21b0111001 | 1110011100 |
| *Phalanger* | 1001000a10 | 100010101d | 1111010001 | 0101100011 | 2a00111001 | 1110011100 |
| *Petaurus* | 1101011110 | 10000-1101 | 1111010021 | 0101100011 | 2a001a1001 | 1000011100 |
| *Pseudochirops* | 1002010110 | 0100101021 | 1110010001 | 0101200011 | 2100111001 | 1110001100 |
| *Cercartetus* | 0000011?10 | 1?000-?111 | 1111010011 | 0101100002 | 211?111001 | 1??0?1110? |
| *Macropus* | 1002000010 | 0000101022 | 1111010101 | 00??300021 | 2010111111 | 1010001100 |
| *Dendrolagus* | 1001010011 | 01a0101022 | 1111010121 | 0001300021 | 2120111011 | 1110011100 |
| *Dorcopsis* | 1002000110 | 0000101022 | 1110000121 | 0001300020 | 2100111111 | 1110001100 |
| *Thylogale* | 1002010110 | 0000101022 | 1111010021 | 0001300021 | 2020111111 | 1010001100 |
| *Vombatus* | a001000100 | 10000-1112 | 1111010010 | 0001200012 | 2a20111111 | 1010011100 |
| *Phascolarctos* | 1002000100 | 10000-0022 | 1110010010 | 0001100011 | 2100101001 | 1110011100 |
| *Asiatherium* | ?????????? | ?????????? | ????????0? | ?????0???? | ???01?1?0? | ?????1???1 |
| *Herpetotherium* | ?????????? | ?????????? | 1?????0??? | ?????????2 | 1?111?1001 | ?1000????? |
| *Mimo/peradectes* | ?????????? | ?????????? | ?????????1 | ?????????? | ??????1??? | ?????????? |
|  |  |  |  |  |  |  |
|  | 61 | 71 | 81 | 91 | 101 | 111 |
|  | | | | | | | | | | | | |
| *Ornithorhynchus* | 200??011?a | 0101100101 | 011011001- | 0000000-00 | a??1?000-1 | 01010-0000 |
| *Tachyglossus* | 000011010a | 0101100101 | 01101a001- | 2011100-00 | 00000010-1 | 01220-0000 |
| *Vincelestes* | ???01??1?0 | 1?11?001?? | 0???110111 | 2000000-00 | 1100001110 | -1110-0000 |
| *Ukhaatherium* | ?????????0 | 1011?001?? | ?10??1???? | 1110011010 | 1101101101 | 01?2100?00 |
| *Asioryctes* | 210???11?? | ?????????? | 0???1?0?0? | 11???11010 | ???11??11? | ?1?010??0? |
| *Deltatheridium* | ?????????? | ?????????? | ?????????? | ?????????? | ???????1?0 | -???0-???? |
| *Mayulestes* | ?????????1 | 101??011?? | 1???110101 | 000?101100 | 11000?1111 | 0111000010 |
| *Pucadelphys* | ?????????1 | 1011000100 | 100?110101 | 0001101100 | 01000?1111 | 0111000010 |
| *Andinodelphys* | 2???0??101 | 1011?01100 | 100?11???? | ?001101100 | 01000?1111 | ?111000?10 |
| *Didelphis* | 2110111100 | 101110a101 | 1100110211 | 2110001100 | 010110a100 | –112110010 |
| *Monodelphis* | 2110111100 | 101110a101 | 1102110211 | 1110001100 | 0101001100 | –101110010 |
| *Caenolestes* | 2110100100 | 1011101101 | 1101110200 | 0110001100 | 0101001100 | –112110010 |
| *Dasyuroides* | 202010110? | 1011?00101 | 1101010200 | 2111101100 | 0101100100 | –0--110012 |
| *Dasyurus* | 2020101101 | 1011100101 | 1102110211 | 2111101100 | 1101101100 | –101110112 |
| *Phascogale* | 2020101101 | 1011100101 | 1101110211 | 2111101100 | 1101100100 | –101110112 |
| *Notoryctes* | 20???001?0 | 0011100111 | 110011200- | 0110101100 | 0101100100 | -0--111-12 |
| *Echymipera* | 2011000000 | 1011001010 | 100001000- | 2010021100 | 0001110102 | –0--110011 |
| *Perameles* | 201110000? | 1011001010 | 100001000- | 2011021100 | 0011110102 | –0--110001 |
| *Dromiciops* | 2020100102 | 1011100101 | 110?110211 | 2110001100 | 0100000110 | –102100012 |
| *Trichosurus* | 2000101100 | 1011101101 | 1101110211 | 2110101100 | 0100100100 | –101110012 |
| *Phalanger* | 1000101100 | 1011100101 | 1101100211 | 2110001100 | 0100101100 | -101111-12 |
| *Petaurus* | 1100001101 | 1011000101 | 1101110211 | 2110101100 | 0100000100 | -101101-12 |
| *Pseudochirops* | 1100??1100 | 1011100101 | 1102100211 | 2110001100 | 0100101100 | -101111-12 |
| *Cercartetus* | 1?00101100 | 1011?00101 | 1100110211 | 2110001100 | 0100000100 | -112101-12 |
| *Macropus* | 0000001112 | 1111111111 | 0100112201 | 0001111101 | 0111100201 | 1102110110 |
| *Dendrolagus* | 0100000111 | 1111101111 | 0100110201 | 1000011101 | 0101100210 | –102110002 |
| *Dorcopsis* | 0000000112 | 1111111110 | 0100112201 | 0001111101 | 0111100201 | 1102110010 |
| *Thylogale* | 0?00001112 | 1111111111 | 0100112201 | 0001011101 | 0111100201 | 1102110110 |
| *Vombatus* | a000101102 | 1011101101 | 0101100211 | 1110011100 | 0101111110 | –0--100012 |
| *Phascolarctos* | 0000001110 | 1011101101 | 1101002211 | 0110011100 | 0101111110 | –0--100012 |
| *Asiatherium* | ?00??????0 | 1??1?0?1?? | ?10??1???? | ?????????? | ?????????? | ?????????? |
| *Herpetotherium* | ?????????1 | 1?1??00101 | 110?110201 | 0010001100 | 0101011112 | ?1110-0?10 |
| *Mimo/peradectes* | ?????????? | ?????????? | ?????1???? | ?????????? | ?????????? | ?????????? |
|  |  |  |  |  |  |  |
|  | 121 | 131 | 141 | 151 | 161 | 171 |
|  | | | | | | | | | | | | |
| *Ornithorhynchus* | 0110100000 | 0102110101 | 000100542? | ??-?????00 | 0----00--- | -0?000??11 |
| *Tachyglossus* | 11001?0100 | 0101110101 | 000102542? | ??-?????00 | 0----00--- | -0?000??11 |
| *Vincelestes* | 01101????? | ?????????? | ??????1310 | 0c?00100?? | ?010121000 | 0??000?000 |
| *Ukhaatherium* | 0??0?????? | ?????????? | ??????0010 | 000??1???? | ?000?21??0 | 00?0000??? |
| *Asioryctes* | ???0?????? | ?1???????1 | 0?????0010 | 0000011??? | ?0?0121000 | 000?00???0 |
| *Deltatheridium* | ?11??????? | ?????????? | ??????1100 | 0001011?12 | 20?0121010 | 01?010???0 |
| *Mayulestes* | 011010?000 | ?????????? | ??????0000 | 0201012012 | 2000121110 | 010010?110 |
| *Pucadelphys* | 0110101000 | ?????????? | ??????0000 | 0211112012 | 2010121110 | 0100100011 |
| *Andinodelphys* | 011010?000 | ????100100 | 0?011?0000 | 0211012012 | 2000020110 | 0?00100011 |
| *Didelphis* | 00-1111000 | 0101101101 | 1100000000 | 0210112112 | 2010121110 | 0100100011 |
| *Monodelphis* | 00-1110000 | 0101101101 | 1101000000 | 0210112112 | 2010121110 | 0100100111 |
| *Caenolestes* | 01001?0011 | 000110?100 | 0001101001 | 1101112212 | 21201211-0 | 0100100111 |
| *Dasyuroides* | 00-0011001 | 0001100110 | 0001201100 | 0210012012 | 2000121110 | 0100100101 |
| *Dasyurus* | 0??0010001 | 0001100110 | 0001201100 | 0210012012 | 2010121110 | 0100100101 |
| *Phascogale* | 0??0011001 | 0001100100 | 0001201100 | 0210012012 | 2020121110 | 0100100101 |
| *Notoryctes* | 11100?1301 | 001?111?11 | 0000022100 | 02-001?0?2 | 2010021100 | 0100000001 |
| *Echymipera* | 1110000100 | 0112100011 | 0010021100 | 0211112112 | 2110121110 | 0110100111 |
| *Perameles* | 1110001100 | 0112a00010 | 0010020101 | 0211112112 | 2110121110 | 0111100111 |
| *Dromiciops* | 00-01?1021 | 010110?101 | 1000000000 | 0100112012 | 2110121100 | 0110100001 |
| *Trichosurus* | 00-01?1021 | 0101100101 | 1010002201 | 1001102110 | 01210111-1 | 010010110a |
| *Phalanger* | 00-01?1021 | 0101100101 | 1010002201 | 1001102111 | 11210111-1 | 010010110a |
| *Petaurus* | 00-0001021 | 0001100101 | 1010002201 | 0101102211 | 11010111-0 | 011010010a |
| *Pseudochirops* | 00-0001021 | 0101100001 | 1010002301 | 0011101110 | 21211011-0 | 1100100101 |
| *Cercartetus* | 01100?1021 | 0001100101 | 1010002301 | 0001102111 | 11211111-0 | 01001001?1 |
| *Macropus* | 1120011200 | 11000-01a2 | --10322301 | 1101102102 | 2101000?-1 | 0100101111 |
| *Dendrolagus* | 1120010200 | 01000-0102 | --10312301 | 1101102102 | 21110001-1 | 0100101111 |
| *Dorcopsis* | 1120011200 | 11000-0112 | --10322301 | 1101102102 | 21a10001-1 | 0100101111 |
| *Thylogale* | 1120011200 | 11000-0112 | --10322301 | 1101102102 | 21010001-1 | 0100101111 |
| *Vombatus* | 0??01?1001 | 0101100101 | 1010004301 | 1101101?00 | 00-1000?-0 | 1101111110 |
| *Phascolarctos* | 0??01?1001 | 11011001?1 | 1010002301 | 0111102200 | 00211001-0 | 1101011111 |
| *Asiatherium* | ?????????? | ?????????? | ?????????? | 00011?2??? | ???01?1??0 | 0??0?0???? |
| *Herpetotherium* | 01101????? | ?????????? | 0?????0000 | 0210112212 | 2000120110 | 0??0100011 |
| *Mimo/peradectes* | ?????????? | ?????????? | ???????000 | 0200010?1? | 2??0121110 | 0?00?00??? |
|  |  |  |  |  |  |  |
|  | 181 | 191 | 201 | 211 | 221 | 231 |
|  | | | | | | | | | | | | |
| *Ornithorhynchus* | 0000000?01 | 0000?0110? | 00000?1000 | 0010301000 | 1000011000 | -000-20200 |
| *Tachyglossus* | 0000000?01 | 0200?0110? | 00000?1000 | 0010401010 | -0000?1000 | -100-20202 |
| *Vincelestes* | 10?0?00??? | 0000100100 | 1200???0?? | ????????00 | 00?0000000 | -000-20000 |
| *Ukhaatherium* | ???1??11?? | ?????000?? | ???0?????? | ?????????3 | ?????????? | ???0-???0? |
| *Asioryctes* | 00110011?? | 3?00000000 | 0110?????? | ?????????3 | ???2??10?1 | 01?0???1?2 |
| *Deltatheridium* | 1??1??10?? | ?0????0001 | 1??0?????? | ????????11 | 0002010001 | 0100--01?1 |
| *Mayulestes* | 10?10010?? | 1000010001 | 12?0?????? | ?????????1 | ?0?2???111 | 0100-?0111 |
| *Pucadelphys* | 10010010?? | 1000010000 | 1200???0?? | ????????1c | 1001001111 | 0110-00101 |
| *Andinodelphys* | 00?10010?? | 101?010000 | 1200???0?? | ????????1c | 000?011111 | 0110-00101 |
| *Didelphis* | 0101001001 | c011100021 | 1c000?0010 | 0001141012 | 1002111001 | 0101010111 |
| *Monodelphis* | 0101001001 | c011000021 | 12000??110 | 0001041012 | 1002101001 | 0101010111 |
| *Caenolestes* | 0201001001 | 1010?01021 | 1100000000 | 0101021002 | 1102001001 | 0101000102 |
| *Dasyuroides* | 0211001001 | 10110000b1 | 01000?0000 | ???0030?12 | ?112?01001 | 0101101102 |
| *Dasyurus* | 0211001001 | 1011000021 | a200010000 | a10?0300?? | ???2??10?1 | 01?111?1?2 |
| *Phascogale* | 0211001001 | 101??000?1 | 0?000??000 | ?????30?12 | 1112101001 | 0101101102 |
| *Notoryctes* | 0201001000 | 2011?0000? | 0?001??000 | 1?002100?? | ???????0?? | ????0????? |
| *Echymipera* | 0211001001 | 2011a01020 | 1100??000? | ?????3???2 | 0002?01001 | 0101120?02 |
| *Perameles* | 0211001001 | 2011a01020 | 12000?0000 | a000231012 | 1002101001 | 0101120102 |
| *Dromiciops* | 0201001010 | 20a1?00020 | 1200121110 | 0100021012 | 1112111001 | 1101101102 |
| *Trichosurus* | 1321111110 | 2010000021 | 120012?a11 | 11001111?2 | 0102111001 | 0101120122 |
| *Phalanger* | 1321111110 | 21c0000021 | 1200121111 | ???0?21??2 | 0102111001 | 0100?20122 |
| *Petaurus* | 0321111110 | 1010?00000 | 1200121111 | 12001211?? | ???????0?? | ?????????? |
| *Pseudochirops* | 1321111110 | 2010?00001 | 120012?111 | 12?01211?? | ???????0?? | ????0????? |
| *Cercartetus* | ?321111110 | 203?000021 | 11001???1? | 1??0121??? | ???????0?? | ?????????? |
| *Macropus* | 1321001110 | 21201000b1 | 1201121110 | 12001211?2 | 1102111001 | 0101121122 |
| *Dendrolagus* | 1321001110 | 21200000b1 | 1201?????0 | ?????2???? | ???????0?? | ?????????? |
| *Dorcopsis* | 1321001110 | 2120?00021 | 1201?????? | ?????2???? | ???????0?? | ?????????? |
| *Thylogale* | 1321001110 | 2120000021 | ac01????10 | 1?0?1211?? | ???????0?? | ????02???? |
| *Vombatus* | 1a21001210 | 302100001? | 11001100a0 | ?2102111?? | ???????0?? | ???0?????? |
| *Phascolarctos* | 1201001110 | 2111?00011 | 12001??000 | ??102111?? | ???????0?? | ???0?????? |
| *Asiatherium* | ???????0?? | c???????0? | 1??????0?? | ?????????? | ?????????1 | 1??10?0??1 |
| *Herpetotherium* | ?2?1????01 | 2?????002? | 1??0?????? | ????????02 | 1102111001 | 0101000111 |
| *Mimo/peradectes* | 01??????01 | 101??0???? | ?????--0-- | ????????12 | 1002111001 | 0101120111 |
|  |  |  |  |  |  |  |
|  |  |  |  |  |  |  |
|  | 241 | 251 |  |  |  |  |
|  | | | | |  |  |  |  |
| *Ornithorhynchus* | 0-0-00-010 | 010-000000 |  |  |  |  |
| *Tachyglossus* | 0-0-00-010 | 010-100000 |  |  |  |  |
| *Vincelestes* | 0-0000-000 | 0000000000 |  |  |  |  |
| *Ukhaatherium* | 10???????? | ???101??10 |  |  |  |  |
| *Asioryctes* | ???0???1?? | ?11101?000 |  |  |  |  |
| *Deltatheridium* | 100000-000 | 0001110000 |  |  |  |  |
| *Mayulestes* | 10001101?? | ???1111001 |  |  |  |  |
| *Pucadelphys* | 100011000a | 11a1111101 |  |  |  |  |
| *Andinodelphys* | 1000110000 | 111111110? |  |  |  |  |
| *Didelphis* | 1000111010 | 0001110010 |  |  |  |  |
| *Monodelphis* | 1000111010 | 0001110010 |  |  |  |  |
| *Caenolestes* | 1100011001 | 0111110010 |  |  |  |  |
| *Dasyuroides* | 111101?1?? | ?111110010 |  |  |  |  |
| *Dasyurus* | 1?11???1?? | ?a1111??10 |  |  |  |  |
| *Phascogale* | 1110012101 | 1111110010 |  |  |  |  |
| *Notoryctes* | ???????1?? | ?????1???0 |  |  |  |  |
| *Echymipera* | 100001?1?? | 0111110010 |  |  |  |  |
| *Perameles* | 1100012100 | 0111110010 |  |  |  |  |
| *Dromiciops* | 1110?121?1 | ?111110010 |  |  |  |  |
| *Trichosurus* | 1?0011101? | ??11110010 |  |  |  |  |
| *Phalanger* | 1100111100 | 0111110010 |  |  |  |  |
| *Petaurus* | ?????????? | ????11???0 |  |  |  |  |
| *Pseudochirops* | ??0????1?? | ?1??11???0 |  |  |  |  |
| *Cercartetus* | ?????????? | ????11???0 |  |  |  |  |
| *Macropus* | 1100111110 | 0111110010 |  |  |  |  |
| *Dendrolagus* | ?????????? | ????11???0 |  |  |  |  |
| *Dorcopsis* | ?????????? | ?????1???0 |  |  |  |  |
| *Thylogale* | ??0????1?? | ?11?11???0 |  |  |  |  |
| *Vombatus* | ??0????1?? | ????11???0 |  |  |  |  |
| *Phascolarctos* | ??0??????? | ????11???0 |  |  |  |  |
| *Asiatherium* | 100??????? | ???1??0??0 |  |  |  |  |
| *Herpetotherium* | 1100111110 | 0111110011 |  |  |  |  |
| *Mimo/peradectes* | 1000011000 | 000111001a |  |  |  |  |
