# Supplementary material for: Cranial Anatomy of the Earliest Marsupials and the Origin of Opossums
Source: PLoS One. 2009 Dec 16;4(12):e8278. doi: 10.1371/journal.pone.0008278 (PMC2789412; doi:10.1371/journal.pone.0008278)
Supplement: Table S3 — List of unambiguous synapomorphies (0.25 MB DOC) [file pone.0008278.s006.doc]

| Node | Character | Steps | CI | Change |
| --- | --- | --- | --- | --- |
| 63 | 3 (HS4 Atl.post.extent tr.proc.) | 1 | 1.00 | 1 ==> 0 |
|  | 5 (HS6 At.trv.proc.vent.to at.f.) | 1 | 0.33 | 1 ==> 0 |
|  | 9 (HS10 Axis post.sp.proc. extent) | 1 | 0.33 | 0 ==> 1 |
|  | 21 (HS22 Articul.among cerv.vert.) | 1 | 1.00 | 0 ==> 1 |
|  | 23 (HS24 T1 transverse process) | 1 | 1.00 | 0 ==> 1 |
|  | 26 (HS27 1st thor.vert.prezyg.lat.) | 2 | 0.40 | 0 ==> 2 |
|  | 29 (HS30 Lumb.3rd.from last metap.) | 1 | 0.37 | 2 ==> 0 |
|  | 35 (HS36 Scapula coracoid process) | 1 | 0.33 | 0 ==> 1 |
|  | 36 (HS37 Acromion vent.extension) | 1 | 0.50 | 1 ==> 0 |
|  | 46 (HS47 Hum.olec.fossa or foramen) | 1 | 0.62 | 0 ==> 1 |
|  | 49 (HS50 Hum.delt.crest extension) | 1 | 0.37 | 1 ==> 0 |
|  | 50 (HS51 Humerus trochlea) | 1 | 1.00 | 0 ==> 1 |
|  | 54 (HS55 Hum head shape) | 1 | 1.00 | 1 ==> 0 |
|  | 57 (HS58 Ulna prox.epiph.articul.) | 1 | 0.50 | 0 ==> 1 |
|  | 58 (HS59 Ulna coronoid process) | 1 | 1.00 | 0 ==> 1 |
|  | 71 (HS72 Obturator foramen size) | 1 | 0.50 | 0 ==> 1 |
|  | 73 (HS75 Acetabular notch) | 1 | 1.00 | 0 ==> 1 |
|  | 88 (HS91 Tib.PS beyond ATim post.) | 1 | 0.50 | 0 ==> 1 |
|  | 108 (HS111 Cal.sus.facet on sustent.) | 1 | 1.00 | 0 ==> 1 |
|  | 147 (HS150 NumberUpperIncisors) | 4 | 0.38 | 5 ==> 1 |
|  | 148 (HS151 Number lower incisors) | 1 | 0.36 | 4 ==> 3 |
|  | 149 (HS152 Number of upper molars) | 1 | 1.00 | 2 ==> 1 |
|  | 166 (HS169 Lower C) | 2 | 0.40 | 0 ==> 2 |
|  | 167 (HS170 Upper C) | 1 | 0.25 | 0 ==> 1 |
|  | 180 (HS183 Maxillo-frontal contact) | 1 | 0.57 | 1 ==> 0 |
|  | 197 (HS200 Shape of nasals) | 1 | 0.33 | 1 ==> 0 |
|  | 202 (HS205 Hypoglossal for.count) | 2 | 0.40 | 0 ==> 2 |
|  | 249 (LA32imprint of transv. sinus bif.) | 1 | 0.25 | 1 ==> 0 |
| 62 | 11 (HS12 Ax.ant.fac.& dens connect) | 1 | 0.33 | 2 ==> 1 |
|  | 89 (HS92 Tibia distal spiral) | 1 | 0.20 | 1 ==> 0 |
|  | 97 (HS100 Astragalar neck) | 1 | 1.00 | 0 ==> 1 |
|  | 148 (HS151 Number lower incisors) | 3 | 0.36 | 3 ==> 0 |
|  | 184 (HS187 Postglenoid process) | 1 | 1.00 | 0 ==> 1 |
|  | 187 (HS190 Postglenoid foramen) | 1 | 1.00 | 0 ==> 1 |
|  | 198 (HS201 Septomaxilla) | 1 | 1.00 | 1 ==> 0 |
|  | 224 (LA6wi108.ant.lam. exp. on lat. brainc.) | 1 | 1.00 | 0 ==> 2 |
|  | 230 (LA12wi122. epitympanic wing) | 1 | 1.00 | 0 ==> 1 |
|  | 241 (LA23caudal tympanic process of petrosal) | 1 | 1.00 | 0 ==> 1 |
|  | 256 (LA39wi129 HoSa 222. Cochlear coiling) | 1 | 1.00 | 0 ==> 1 |
| 34 | 92 (HS95 Asnav.fac.cont.medially) | 1 | 0.25 | 0 ==> 1 |
|  | 96 (HS99 Asfib.facet-ATil angle) | 1 | 0.50 | 0 ==> 1 |
|  | 99 (HS102 As.sust.fac.medial reach) | 1 | 1.00 | 0 ==> 1 |
|  | 105 (HS108 Atil-ridge-Afib) | 1 | 0.25 | 0 ==> 1 |
|  | 115 (HS118 Cal.sus.fac.medlat.orien.) | 1 | 0.50 | 0 ==> 1 |
| 61 | 149 (HS152 Number of upper molars) | 1 | 1.00 | 1 ==> 0 |
|  | 154 (HS157 Metaconule) | 1 | 0.20 | 0 ==> 1 |
|  | 169 (HS172 Lower I2 staggered) | 1 | 0.33 | 0 ==> 1 |
|  | 172 (HS175 Marsup.dental replace.) | 1 | 1.00 | 0 ==> 1 |
|  | 175 (HS178 Ang.proc.med.inflexion) | 1 | 0.33 | 0 ==> 1 |
|  | 200 (HS203 Premax.palatal process) | 1 | 0.20 | 0 ==> 1 |
|  | 255 (LA38wi147. sulcus for stapedial artery) | 1 | 0.50 | 0 ==> 1 |
| 60 | 157 (HS160 Crid.obli.-trid.intersec.) | 1 | 0.33 | 1 ==> 2 |
| 59 | 44 (HS45 Humerus capitulum) | 1 | 0.20 | 0 ==> 1 |
|  | 152 (HS155 Up.M2 parac.-metac.size) | 2 | 0.33 | 0 ==> 2 |
| 36 | 10 (HS11 Ax.extra pair trv.proc.) | 1 | 0.50 | 0 ==> 1 |
|  | 95 (HS98 As.ampt visible dorsally) | 1 | 0.12 | 0 ==> 1 |
|  | 196 (HS199 Squamosal med.process) | 1 | 1.00 | 0 ==> 1 |
|  | 228 (LA10wi148, HoSa220. deep gr.for int.car.a) | 1 | 1.00 | 0 ==> 1 |
|  | 229 (LA11Deep & large fossa for tens.tymp.m.) | 1 | 1.00 | 0 ==> 1 |
|  | 257 (LA45Tymp.sin.dans lat.trough du periotic) | 1 | 1.00 | 0 ==> 1 |
| 35 | 41 (HS42 Neck infra.suprasp.width) | 1 | 0.25 | 1 ==> 2 |
|  | 153 (HS156 Up.M2 centrocrista shape) | 1 | 0.16 | 0 ==> 1 |
|  | 200 (HS203 Premax.palatal process) | 1 | 0.20 | 1 ==> 0 |
|  | 233 (LA15Broad shelf of bone surr.fen.cochl.) | 1 | 1.00 | 0 ==> 1 |
|  | 258 (LA47Tympanic sinus elements = pe + sq) | 1 | 1.00 | 0 ==> 1 |
| 58 | 154 (HS157 Metaconule) | 1 | 0.20 | 1 ==> 0 |
|  | 158 (HS161 Up.I arcade shape) | 1 | 0.25 | 0 ==> 1 |
|  | 199 (HS202 Palatal vacuities) | 2 | 0.53 | 0 ==> 2 |
|  | 220 (LA2wi109. Cavum epiptericum floored by) | 1 | 1.00 | 1 ==> 2 |
|  | 225 (LA7anterior lamina) | 1 | 0.50 | 0 ==> 1 |
|  | 259 (LA48Tymp.sin.elements=al+pe ou al exclus.) | 1 | 0.50 | 0 ==> 1 |
| 57 | 92 (HS95 Asnav.fac.cont.medially) | 1 | 0.25 | 0 ==> 1 |
|  | 109 (HS112 Cal.sustentaculum posit.) | 1 | 0.20 | 1 ==> 0 |
|  | 110 (HS113 Cal.ectal facet axis) | 1 | 0.28 | 1 ==> 0 |
|  | 115 (HS118 Cal.sus.fac.medlat.orien.) | 1 | 0.50 | 0 ==> 1 |
|  | 163 (HS166 Size I3/I2) | 1 | 0.25 | 0 ==> 1 |
| 38 | 236 (LA18Tymp.aperture of hiatus Fall.) | 1 | 0.22 | 0 ==> 1 |
|  | 252 (LA35HoSa219. Posttemp.sulc.on squam.surf) | 1 | 0.50 | 1 ==> 0 |
|  | 253 (LA36posttemporal notch/foramen) | 1 | 0.33 | 1 ==> 0 |
| 37 | 153 (HS156 Up.M2 centrocrista shape) | 1 | 0.16 | 0 ==> 1 |
|  | 249 (LA32imprint of transv. sinus bif.) | 1 | 0.25 | 0 ==> 1 |
| 56 | 4 (HS5 Atlas cranial fac.shape) | 1 | 0.25 | 1 ==> 0 |
|  | 22 (HS23 Articulation C4,C5 type) | 1 | 1.00 | 0 ==> 1 |
|  | 130 (HS133 Cucal.facet outer shelf) | 1 | 0.33 | 0 ==> 1 |
|  | 132 (HS135 Nav.-entoc.relationship) | 1 | 0.25 | 1 ==> 0 |
|  | 147 (HS150 NumberUpperIncisors) | 1 | 0.38 | 0 ==> 1 |
|  | 212 (HS224 Parasep.cartilage shape) | 1 | 0.50 | 0 ==> 1 |
|  | 240 (LA22mastoid tympanic process see wi131) | 1 | 0.50 | 1 ==> 2 |
| 55 | 17 (HS18 C5 & T1 body length) | 1 | 0.33 | 0 ==> 1 |
|  | 52 (HS53 Hum med epicondyle size) | 1 | 0.28 | 1 ==> 0 |
|  | 91 (HS94 As.ATim-ATil angle) | 1 | 0.15 | 1 ==> 2 |
|  | 107 (HS110 As.head-sust.fac.cont.) | 1 | 0.28 | 1 ==> 0 |
|  | 113 (HS116 Cal.ant.peron.tub.shape) | 1 | 0.40 | 1 ==> 0 |
|  | 120 (HS123 Cal.sus.fac.-ectal merge) | 2 | 0.40 | 0 ==> 2 |
|  | 179 (HS182 Palatal maxilla L/W) | 1 | 0.25 | 1 ==> 0 |
|  | 235 (LA17Tympanic wing of petrosal) | 1 | 0.20 | 0 ==> 1 |
|  | 248 (LA30wr77, HoSa217. prootic canal) | 1 | 0.20 | 0 ==> 1 |
| 43 | 105 (HS108 Atil-ridge-Afib) | 1 | 0.25 | 0 ==> 1 |
|  | 139 (HS142 Mt V prox.proc.vent.cub.) | 1 | 0.50 | 0 ==> 1 |
|  | 153 (HS156 Up.M2 centrocrista shape) | 1 | 0.16 | 0 ==> 1 |
| 40 | 43 (HS44 Humerus lateral ridge) | 1 | 0.26 | 1 ==> 2 |
|  | 63 (HS64 Scaphoid distolat.process) | 1 | 0.50 | 1 ==> 2 |
|  | 94 (HS97 Asnav.fac.transver.wider) | 1 | 0.20 | 0 ==> 1 |
|  | 126 (HS129 Cal.accessory facet) | 1 | 0.33 | 0 ==> 1 |
|  | 140 (HS143 Mt II or III more prox.) | 1 | 0.40 | 1 ==> 0 |
|  | 145 (HS148 Mt III-Mt I thickness) | 2 | 0.42 | 0 ==> 2 |
|  | 223 (LA5Expan.of cris.petr.that forms a thin l) | 1 | 0.50 | 0 ==> 1 |
|  | 237 (LA19Stylomastoid foramen (see wr54)) | 1 | 0.33 | 0 ==> 1 |
|  | 243 (LA25petrosal plate (see HoSa216)) | 1 | 0.50 | 0 ==> 1 |
| 39 | 53 (HS54 Hum distal end size) | 1 | 0.20 | 1 ==> 0 |
|  | 89 (HS92 Tibia distal spiral) | 1 | 0.20 | 0 ==> 1 |
|  | 101 (HS104 Astragalar canal) | 1 | 0.40 | 0 ==> 1 |
|  | 118 (HS121 Calc sust facet post convex) | 1 | 0.50 | 0 ==> 1 |
| 42 | 24 (HS25 T1 trv.process level) | 1 | 0.14 | 1 ==> 0 |
|  | 29 (HS30 Lumb.3rd.from last metap.) | 1 | 0.37 | 0 ==> 2 |
|  | 35 (HS36 Scapula coracoid process) | 1 | 0.33 | 2 ==> 3 |
|  | 47 (HS48 Hum.laminar supinat.crest) | 1 | 1.00 | 1 ==> 0 |
|  | 79 (HS81Fib.med.proximal shelf) | 1 | 0.50 | 0 ==> 1 |
|  | 88 (HS91 Tib.PS beyond ATim post.) | 2 | 0.50 | 2 ==> 0 |
|  | 121 (HS124 Cal.sus.facet ant. edge) | 1 | 0.33 | 0 ==> 1 |
|  | 133 (HS136 Nav.shelf btwn.cub & as.) | 1 | 1.00 | 0 ==> 1 |
|  | 146 (HS149 Foot ungual digit IV) | 2 | 0.33 | 0 ==> 2 |
|  | 215 (HS227 Pouch type) | 1 | 0.66 | 0 ==> 2 |
| 41 | 25 (HS26 1st thor.tall spi.process) | 1 | 0.60 | 0 ==> 1 |
|  | 31 (HS32 Caud.vert.hyper.chevrons) | 1 | 1.00 | 0 ==> 1 |
|  | 36 (HS37 Acromion vent.extension) | 1 | 0.50 | 0 ==> 1 |
|  | 37 (HS38 Scapular medial crest) | 1 | 1.00 | 0 ==> 1 |
|  | 42 (HS43 Humerus medial relief) | 1 | 0.60 | 0 ==> 1 |
|  | 46 (HS47 Hum.olec.fossa or foramen) | 1 | 0.62 | 1 ==> 2 |
|  | 53 (HS54 Hum distal end size) | 1 | 0.20 | 1 ==> 0 |
|  | 68 (HS69 Both McI & V hypereduced) | 1 | 1.00 | 1 ==> 0 |
|  | 75 (HS77 Epipubic bones prox.size) | 1 | 0.33 | 1 ==> 0 |
|  | 77 (HS79 Fem.head-gr.troch.height) | 1 | 0.42 | 0 ==> 1 |
|  | 78 (HS80Femur lesser trochanter) | 1 | 1.00 | 1 ==> 0 |
|  | 80 (HS82Fib.prox.post.extension) | 1 | 0.33 | 1 ==> 0 |
|  | 82 (HS84Femur-fib.articulation) | 1 | 0.50 | 1 ==> 0 |
|  | 85 (HS88 Tibia med.malleolus) | 1 | 0.33 | 1 ==> 0 |
|  | 92 (HS95 Asnav.fac.cont.medially) | 1 | 0.25 | 1 ==> 0 |
|  | 96 (HS99 Asfib.facet-ATil angle) | 2 | 0.50 | 0 ==> 2 |
|  | 102 (HS105 Astragalar post.facet) | 1 | 0.50 | 1 ==> 0 |
|  | 106 (HS109 Atim.shorter) | 1 | 0.33 | 0 ==> 1 |
|  | 110 (HS113 Cal.ectal facet axis) | 2 | 0.28 | 0 ==> 2 |
|  | 120 (HS123 Cal.sus.fac.-ectal merge) | 1 | 0.40 | 2 ==> 1 |
|  | 130 (HS133 Cucal.facet outer shelf) | 1 | 0.33 | 1 ==> 0 |
|  | 132 (HS135 Nav.-entoc.relationship) | 1 | 0.25 | 0 ==> 1 |
|  | 143 (HS146 Syndactyly) | 1 | 0.50 | 0 ==> 1 |
|  | 154 (HS157 Metaconule) | 1 | 0.20 | 0 ==> 1 |
|  | 173 (HS176 Par.alis.vs.squam.fron.) | 1 | 0.33 | 0 ==> 1 |
|  | 179 (HS182 Palatal maxilla L/W) | 1 | 0.25 | 0 ==> 1 |
|  | 197 (HS200 Shape of nasals) | 1 | 0.33 | 0 ==> 1 |
| 54 | 20 (HS21 C7 trv.foramen) | 1 | 0.70 | 0 ==> 1 |
|  | 26 (HS27 1st thor.vert.prezyg.lat.) | 1 | 0.40 | 2 ==> 1 |
|  | 32 (HS33 Prehensile tail) | 1 | 0.33 | 0 ==> 1 |
|  | 35 (HS36 Scapula coracoid process) | 1 | 0.33 | 2 ==> 1 |
|  | 42 (HS43 Humerus medial relief) | 1 | 0.60 | 0 ==> 1 |
|  | 44 (HS45 Humerus capitulum) | 1 | 0.20 | 1 ==> 0 |
|  | 51 (ModifHS52 Hum.capitulum prox.extent) | 1 | 0.50 | 0 ==> 1 |
|  | 89 (HS92 Tibia distal spiral) | 1 | 0.20 | 0 ==> 1 |
|  | 104 (HS107 ATim-ridge-Atil) | 1 | 0.20 | 1 ==> 0 |
|  | 116 (HS119 Cal.sus.antpost.orient.) | 1 | 0.25 | 1 ==> 0 |
|  | 129 (HS132 Cucal.pox-dors.angle) | 1 | 0.40 | 1 ==> 2 |
|  | 141 (HS144 Hallux opposable) | 1 | 0.50 | 0 ==> 1 |
|  | 189 (HS192 Incisura tymp.posit.) | 1 | 1.00 | 0 ==> 1 |
|  | 190 (HS193 Size incisura tympanica) | 1 | 0.50 | 1 ==> 0 |
|  | 205 (HS208 Malleolar neck) | 1 | 0.50 | 0 ==> 1 |
|  | 206 (HS209 Ossicular axis) | 1 | 0.66 | 1 ==> 2 |
|  | 207 (HS210 Manubr-incud.lev.arm rat.) | 1 | 0.33 | 0 ==> 1 |
|  | 208 (HS211 Stapedial ratio) | 1 | 0.50 | 0 ==> 1 |
|  | 209 (HS212 Stapedial foramen) | 1 | 0.50 | 0 ==> 1 |
| 53 | 61 (HS62 Hand lunatum) | 1 | 0.50 | 2 ==> 1 |
|  | 63 (HS64 Scaphoid distolat.process) | 1 | 0.50 | 1 ==> 0 |
|  | 117 (HS120 Cal.sus.fac.antpost.conc.) | 1 | 0.33 | 0 ==> 1 |
|  | 143 (HS146 Syndactyly) | 1 | 0.50 | 0 ==> 1 |
|  | 147 (HS150 NumberUpperIncisors) | 1 | 0.38 | 1 ==> 2 |
|  | 148 (HS151 Number lower incisors) | 1 | 0.36 | 1 ==> 2 |
|  | 150 (HS153 Upper molars shape) | 1 | 0.33 | 0 ==> 1 |
|  | 154 (HS157 Metaconule) | 1 | 0.20 | 0 ==> 1 |
|  | 156 (HS159 Lower molars paraconid) | 1 | 1.00 | 1 ==> 0 |
|  | 160 (HS163 Lower dP2) | 1 | 0.28 | 2 ==> 1 |
|  | 161 (HS164 Upper dP2) | 1 | 0.28 | 2 ==> 1 |
|  | 164 (HS167 procumbent lower I3) | 1 | 1.00 | 0 ==> 1 |
|  | 166 (HS169 Lower C) | 1 | 0.40 | 2 ==> 1 |
|  | 182 (HS185 Tympanic wing of alisph.) | 1 | 0.57 | 2 ==> 3 |
|  | 183 (HS186 Ectotympanic shape) | 1 | 0.28 | 1 ==> 2 |
|  | 185 (HS188 Ext.audit.meatus posit.) | 1 | 0.50 | 0 ==> 1 |
|  | 186 (HS189 Fusion ectotympanic) | 1 | 0.50 | 0 ==> 1 |
|  | 188 (HS191 Postglenoid for.position) | 1 | 0.66 | 0 ==> 1 |
|  | 215 (HS227 Pouch type) | 1 | 0.66 | 0 ==> 1 |
| 52 | 1 (HS1 Atlantal foramen) | 1 | 0.55 | 0 ==> 1 |
|  | 4 (HS5 Atlas cranial fac.shape) | 1 | 0.25 | 0 ==> 1 |
|  | 40 (HS41 Scapular spine width at neck) | 1 | 0.50 | 2 ==> 1 |
| 51 | 7 (HS8 Atlas intercentrum) | 1 | 0.50 | 1 ==> 0 |
|  | 18 (HS19 C6 trv.proc.post.extent) | 1 | 0.20 | 1 ==> 0 |
|  | 52 (HS53 Hum med epicondyle size) | 1 | 0.28 | 0 ==> 1 |
|  | 105 (HS108 Atil-ridge-Afib) | 1 | 0.25 | 0 ==> 1 |
|  | 116 (HS119 Cal.sus.antpost.orient.) | 1 | 0.25 | 0 ==> 1 |
|  | 132 (HS135 Nav.-entoc.relationship) | 1 | 0.25 | 0 ==> 1 |
|  | 181 (HS184 Lacrimal tubercle) | 1 | 0.20 | 0 ==> 1 |
| 50 | 125 (HS128 Cal.sus.fac.ant.extent) | 1 | 0.25 | 0 ==> 1 |
|  | 151 (HS154 Parac.& metac.placement) | 1 | 0.33 | 0 ==> 1 |
|  | 170 (HS173 Bunoloph.or lophodonty) | 1 | 0.50 | 0 ==> 1 |
|  | 177 (HS180 Infraorbital canal bones) | 1 | 1.00 | 0 ==> 1 |
| 49 | 29 (HS30 Lumb.3rd.from last metap.) | 1 | 0.37 | 0 ==> 1 |
|  | 77 (HS79 Fem.head-gr.troch.height) | 1 | 0.42 | 0 ==> 1 |
|  | 117 (HS120 Cal.sus.fac.antpost.conc.) | 1 | 0.33 | 1 ==> 0 |
|  | 249 (LA32imprint of transv. sinus bif.) | 1 | 0.25 | 0 ==> 1 |
| 48 | 32 (HS33 Prehensile tail) | 1 | 0.33 | 1 ==> 0 |
|  | 61 (HS62 Hand lunatum) | 1 | 0.50 | 1 ==> 0 |
|  | 91 (HS94 As.ATim-ATil angle) | 1 | 0.15 | 2 ==> 1 |
|  | 96 (HS99 Asfib.facet-ATil angle) | 1 | 0.50 | 0 ==> 1 |
|  | 104 (HS107 ATim-ridge-Atil) | 1 | 0.20 | 0 ==> 1 |
|  | 129 (HS132 Cucal.pox-dors.angle) | 2 | 0.40 | 2 ==> 0 |
|  | 148 (HS151 Number lower incisors) | 1 | 0.36 | 2 ==> 3 |
|  | 152 (HS155 Up.M2 parac.-metac.size) | 1 | 0.33 | 0 ==> 1 |
|  | 159 (HS162 Upper dP1) | 1 | 0.50 | 1 ==> 0 |
|  | 166 (HS169 Lower C) | 1 | 0.40 | 1 ==> 0 |
|  | 167 (HS170 Upper C) | 1 | 0.25 | 1 ==> 0 |
|  | 179 (HS182 Palatal maxilla L/W) | 1 | 0.25 | 0 ==> 1 |
|  | 185 (HS188 Ext.audit.meatus posit.) | 1 | 0.50 | 1 ==> 0 |
|  | 186 (HS189 Fusion ectotympanic) | 1 | 0.50 | 1 ==> 0 |
|  | 210 (HS213 Bullate stapes) | 1 | 0.50 | 1 ==> 0 |
| 46 | 28 (HS29 Last lumbar for on dors.) | 1 | 0.16 | 0 ==> 1 |
|  | 35 (HS36 Scapula coracoid process) | 1 | 0.33 | 2 ==> 3 |
|  | 39 (HS40 Infraspinous fossa width) | 1 | 0.40 | 1 ==> 2 |
|  | 72 (HS73 Hyperdev.iliopubic proc.) | 1 | 0.50 | 0 ==> 1 |
|  | 79 (HS81Fib.med.proximal shelf) | 1 | 0.50 | 0 ==> 1 |
|  | 84 (HS86 Sesamoids UAJ) | 1 | 0.25 | 1 ==> 0 |
|  | 89 (HS92 Tibia distal spiral) | 1 | 0.20 | 1 ==> 0 |
|  | 92 (HS95 Asnav.fac.cont.medially) | 1 | 0.25 | 1 ==> 0 |
|  | 93 (HS96 Asnav.fac.cont.ventrmed.) | 1 | 0.25 | 1 ==> 0 |
|  | 100 (HS103 Asnav.fac.ant.to trochlea) | 1 | 1.00 | 0 ==> 1 |
|  | 108 (HS111 Cal.sus.facet on sustent.) | 1 | 1.00 | 1 ==> 2 |
|  | 121 (HS124 Cal.sus.facet ant. edge) | 1 | 0.33 | 0 ==> 1 |
|  | 125 (HS128 Cal.sus.fac.ant.extent) | 1 | 0.25 | 1 ==> 0 |
|  | 128 (HS131 Cucal.facet shape) | 1 | 0.75 | 0 ==> 2 |
|  | 130 (HS133 Cucal.facet outer shelf) | 1 | 0.33 | 1 ==> 0 |
|  | 134 (HS137 Nav.-cuboid size) | 1 | 0.66 | 1 ==> 0 |
|  | 135 (HS138 Mesoc.contact with nav.) | 1 | 1.00 | 1 ==> 0 |
|  | 140 (HS143 Mt II or III more prox.) | 1 | 0.40 | 1 ==> 2 |
|  | 145 (HS148 Mt III-Mt I thickness) | 3 | 0.42 | 0 ==> 3 |
|  | 146 (HS149 Foot ungual digit IV) | 1 | 0.33 | 0 ==> 1 |
|  | 160 (HS163 Lower dP2) | 2 | 0.28 | 0 ==> 2 |
|  | 161 (HS164 Upper dP2) | 2 | 0.28 | 0 ==> 2 |
|  | 163 (HS166 Size I3/I2) | 1 | 0.25 | 2 ==> 1 |
|  | 204 (HS207 Masseteric & dental canal) | 1 | 1.00 | 0 ==> 1 |
| 45 | 4 (HS5 Atlas cranial fac.shape) | 1 | 0.25 | 1 ==> 2 |
|  | 48 (HS49 Hum.greater tub.height) | 1 | 0.25 | 0 ==> 1 |
|  | 56 (HS57 Humerus shaft curvature) | 1 | 0.33 | 1 ==> 0 |
|  | 76 (HS78 Medlat.constrict.gr.troc.) | 1 | 1.00 | 0 ==> 1 |
|  | 87 (HS90 Tibia prox.cross section) | 2 | 0.33 | 0 ==> 2 |
|  | 91 (HS94 As.ATim-ATil angle) | 1 | 0.15 | 1 ==> 0 |
|  | 94 (HS97 Asnav.fac.transver.wider) | 1 | 0.20 | 0 ==> 1 |
|  | 103 (HS106 As.post.facet posteriorly subte) | 1 | 0.50 | 0 ==> 1 |
|  | 110 (HS113 Cal.ectal facet axis) | 1 | 0.28 | 0 ==> 1 |
|  | 120 (HS123 Cal.sus.fac.-ectal merge) | 2 | 0.40 | 2 ==> 0 |
|  | 131 (HS134 Cucal.vent.facet) | 1 | 0.50 | 0 ==> 1 |
|  | 139 (HS142 Mt V prox.proc.vent.cub.) | 1 | 0.50 | 0 ==> 1 |
|  | 146 (HS149 Foot ungual digit IV) | 1 | 0.33 | 1 ==> 2 |
| 44 | 42 (HS43 Humerus medial relief) | 1 | 0.60 | 1 ==> 0 |
|  | 52 (HS53 Hum med epicondyle size) | 1 | 0.28 | 1 ==> 0 |
|  | 118 (HS121 Calc sust facet post convex) | 1 | 0.50 | 0 ==> 1 |
| 47 | 9 (HS10 Axis post.sp.proc. extent) | 1 | 0.33 | 1 ==> 0 |
|  | 15 (HS16 C5 trv.proc.two heads) | 1 | 0.25 | 1 ==> 0 |
|  | 30 (HS31 Caudal vertebrae count) | 1 | 1.00 | 1 ==> 0 |
|  | 106 (HS109 Atim.shorter) | 1 | 0.33 | 0 ==> 1 |
|  | 112 (HS115 Cal.ant.peroneal tub.) | 1 | 0.33 | 1 ==> 0 |
|  | 116 (HS119 Cal.sus.antpost.orient.) | 1 | 0.25 | 1 ==> 0 |
|  | 162 (HS165 Upper incisors spatulate) | 1 | 0.25 | 1 ==> 0 |
|  | 170 (HS173 Bunoloph.or lophodonty) | 1 | 0.50 | 1 ==> 0 |
|  | 171 (HS174 Selenodonty developed) | 1 | 0.50 | 0 ==> 1 |
|  | 174 (HS177 Width fron. vs width par.) | 1 | 0.50 | 0 ==> 1 |
|  | 176 (HS179 mandib symphysis fused) | 1 | 1.00 | 0 ==> 1 |
|  | 182 (HS185 Tympanic wing of alisph.) | 1 | 0.57 | 3 ==> 2 |
|  | 194 (HS197 Intramural transv.canal) | 1 | 0.25 | 0 ==> 1 |
|  | 199 (HS202 Palatal vacuities) | 1 | 0.53 | 2 ==> 1 |
|  | 208 (HS211 Stapedial ratio) | 1 | 0.50 | 1 ==> 0 |
|  | 213 (HS225 Parasep.cart.cross sect.) | 1 | 0.50 | 0 ==> 1 |
|  | 215 (HS227 Pouch type) | 1 | 0.66 | 1 ==> 2 |
|  | 234 (LA16rostral tymp.proc.of petr.) | 1 | 0.25 | 1 ==> 0 |
