# Supplementary figures and images for: Cranial Anatomy of the Earliest Marsupials and the Origin of Opossums
Source: PLoS One. 2009 Dec 16;4(12):e8278. doi: 10.1371/journal.pone.0008278 (PMC2789412; doi:10.1371/journal.pone.0008278)

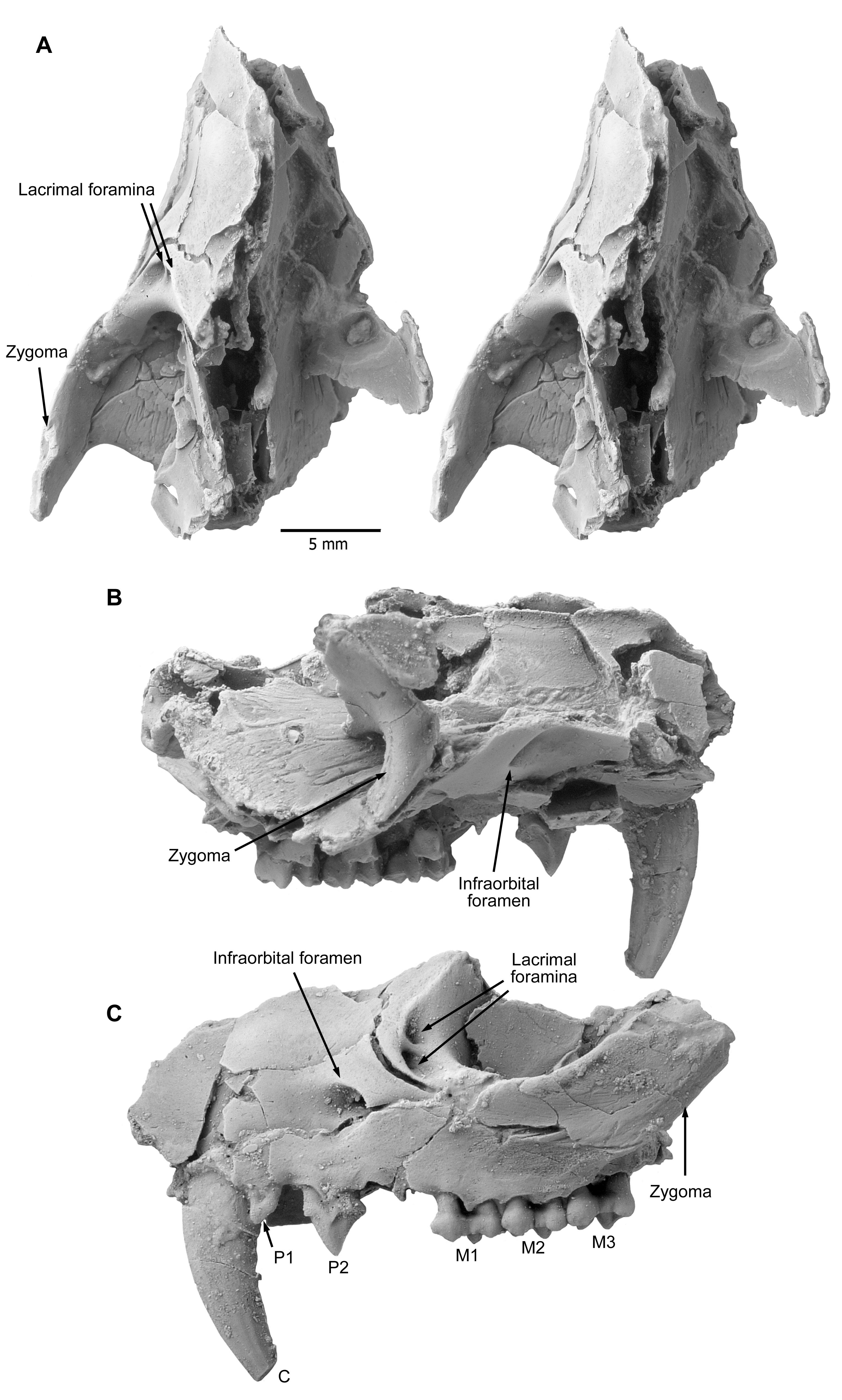

Supplement: Figure S1 — Photographs of anterior portion of the skull of Mimoperadectes houdei in (A) dorsal (stereo photographs), (B) right and (C) left lateral views. Abbreviations: C, canine; P, premolar: M, molar (5.00 MB TIF) [file pone.0008278.s007.tif]

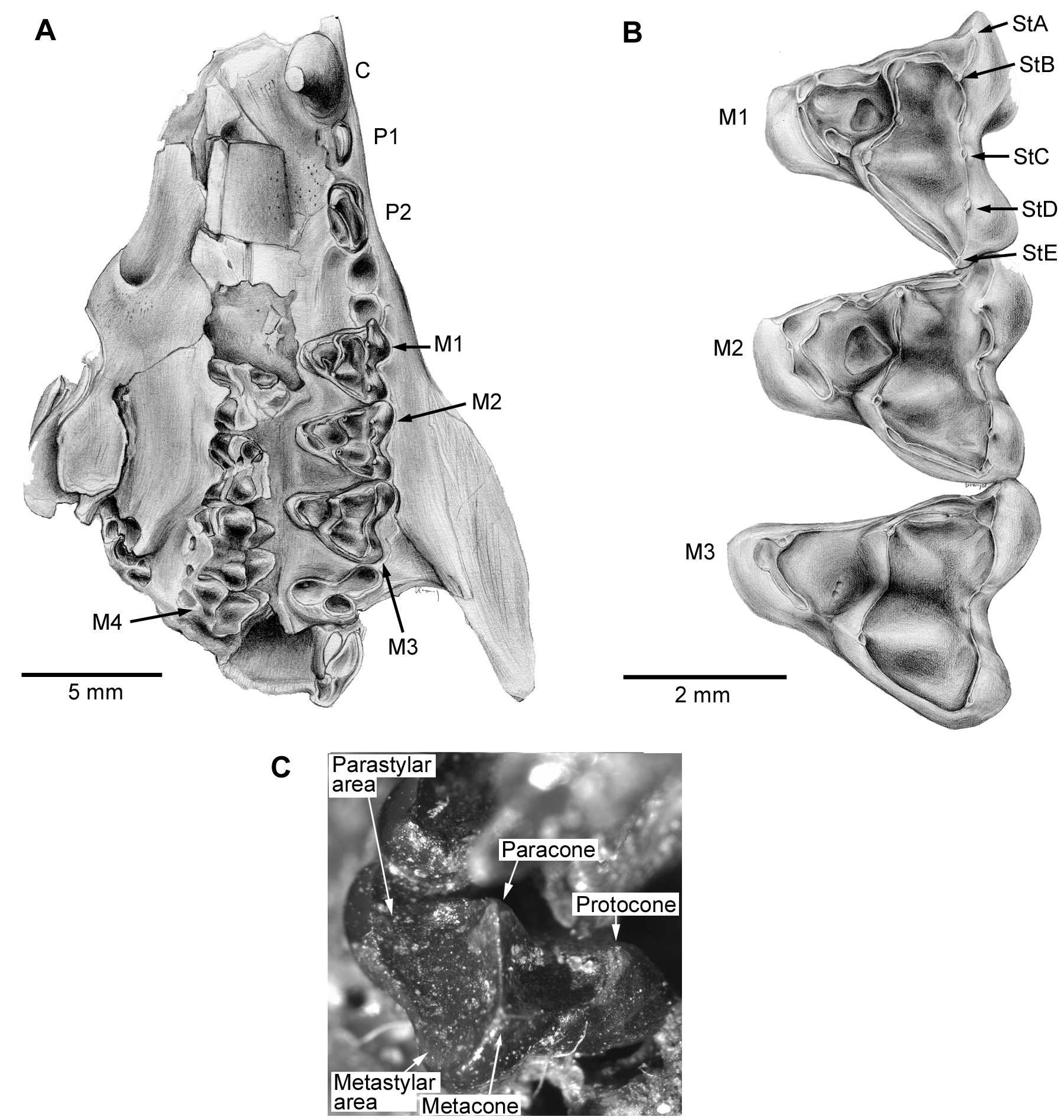

Supplement: Figure S2 — Anterior portion of skull and molars of Mimoperadectes houdei. (A) Drawing of anterior portion of skull in ventral view, at a slight angle from photographs in Figure 1A, (B) drawing of left molars M1-3 of in occlusal view, at a slight angle from photographs in Figure 1B, (C) photograph of right M4 in posterobuccal view. (Displacement of this molar during preservation prevents a straight buccal view and use of a scalebar). Abbreviation: St, stylar cusp (1.39 MB TIF) [file pone.0008278.s008.tif]

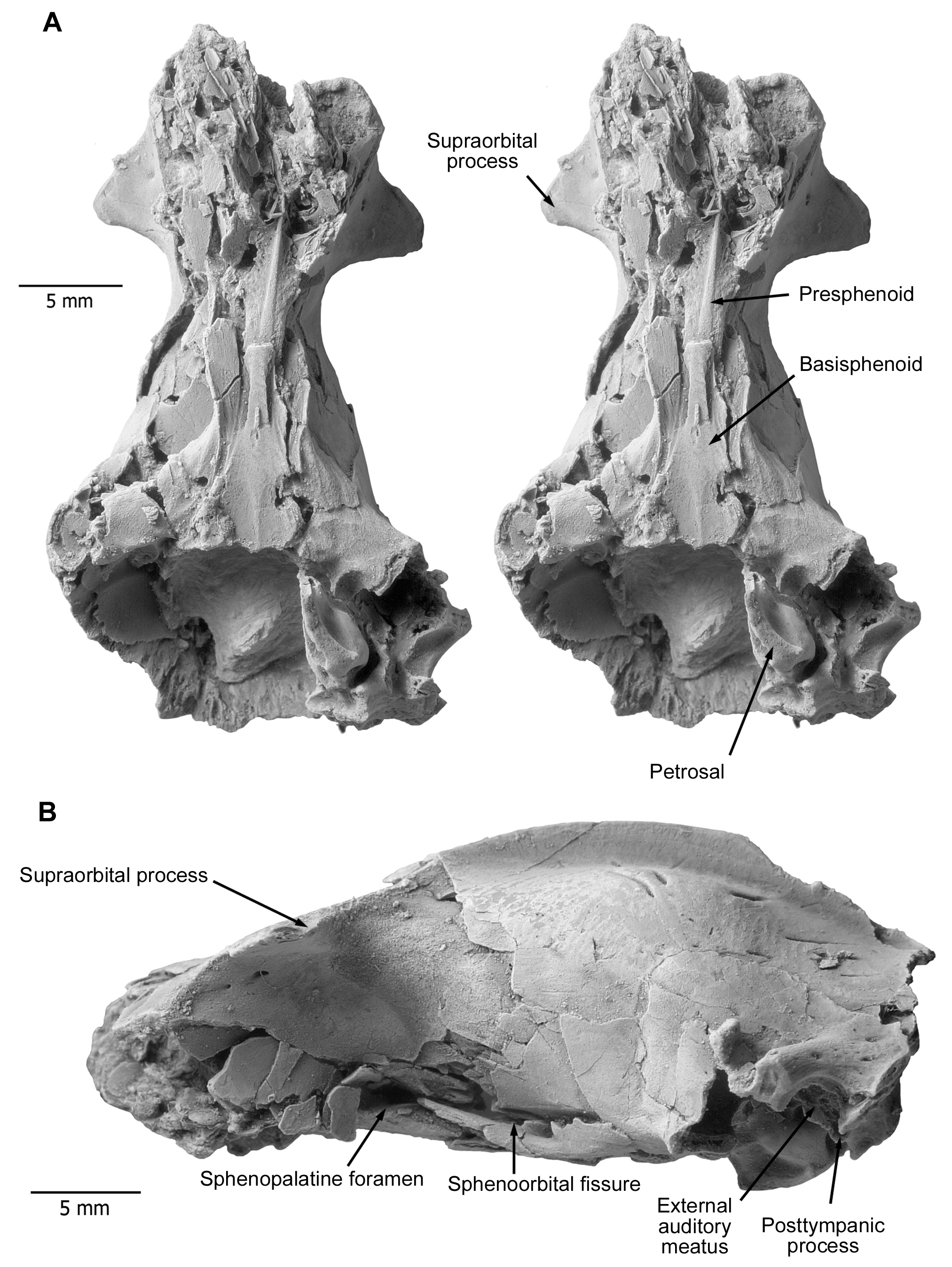

Supplement: Figure S3 — Photographs of posterior portion of the skull of Mimoperadectes houdei in (A) ventral (stereo photographs) and (C) left lateral views (2.40 MB TIF) [file pone.0008278.s009.tif]

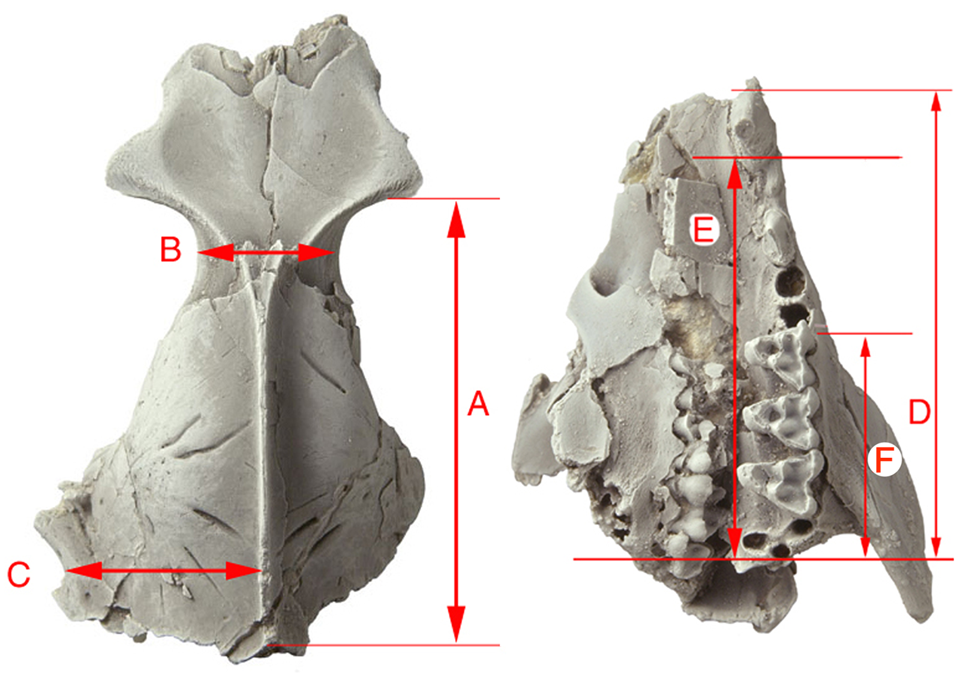

Supplement: Figure S4 — Key to cranial measurements of Mimoperadectes houdei (0.72 MB TIF) [file pone.0008278.s010.tif]

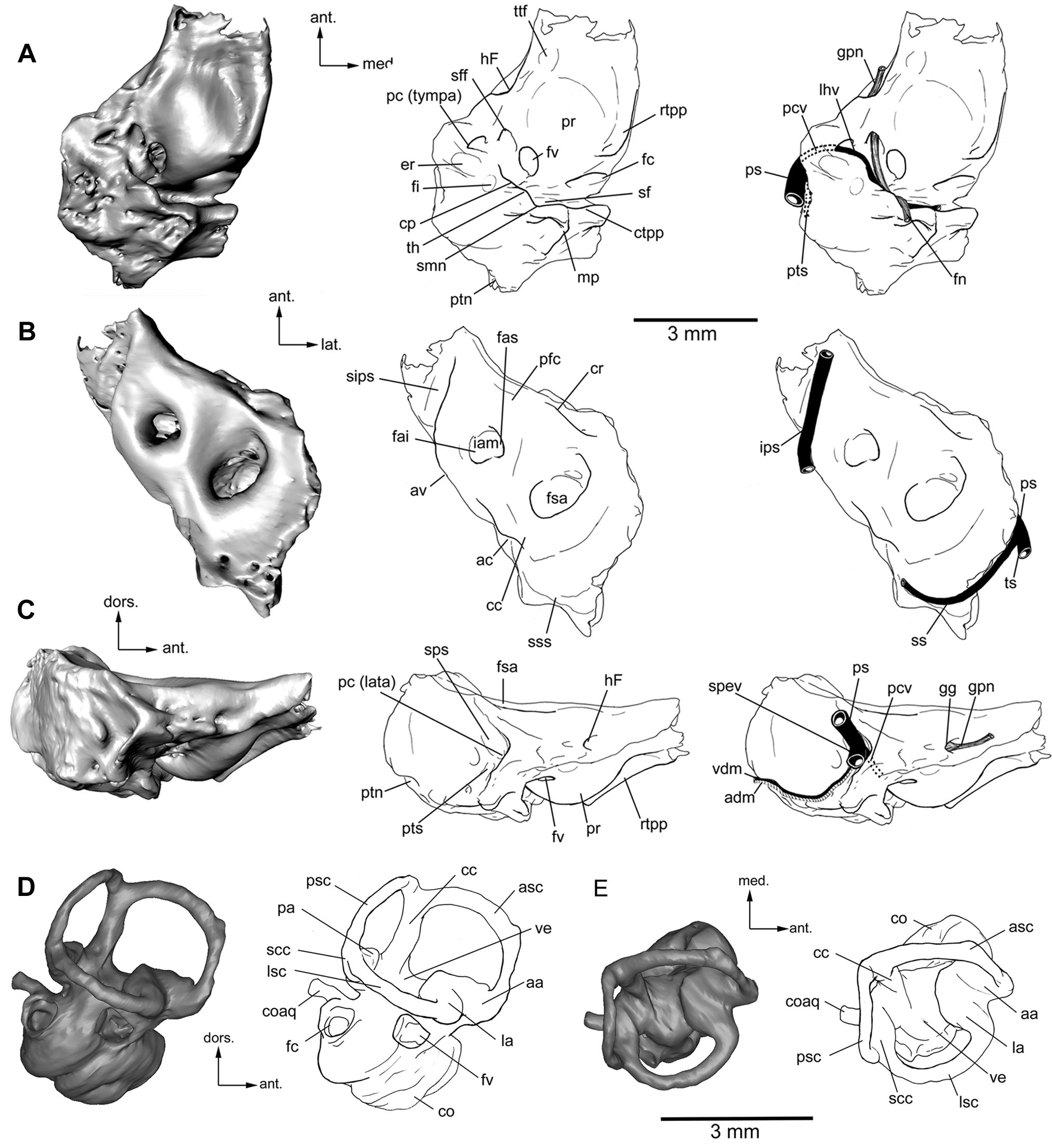

Supplement: Figure S5 — Computer tomography reconstruction (CT) of right petrosal of Mimoperadectes houdei 482355 in ventral (A), dorsal (B), and lateral (C) views (scale above) and anatomical structures of the endocast of the right bony labyrinth in lateral (D) and dorsal (E) views (scale below). See Text S1 for abbreviations (0.95 MB TIF) [file pone.0008278.s011.tif]

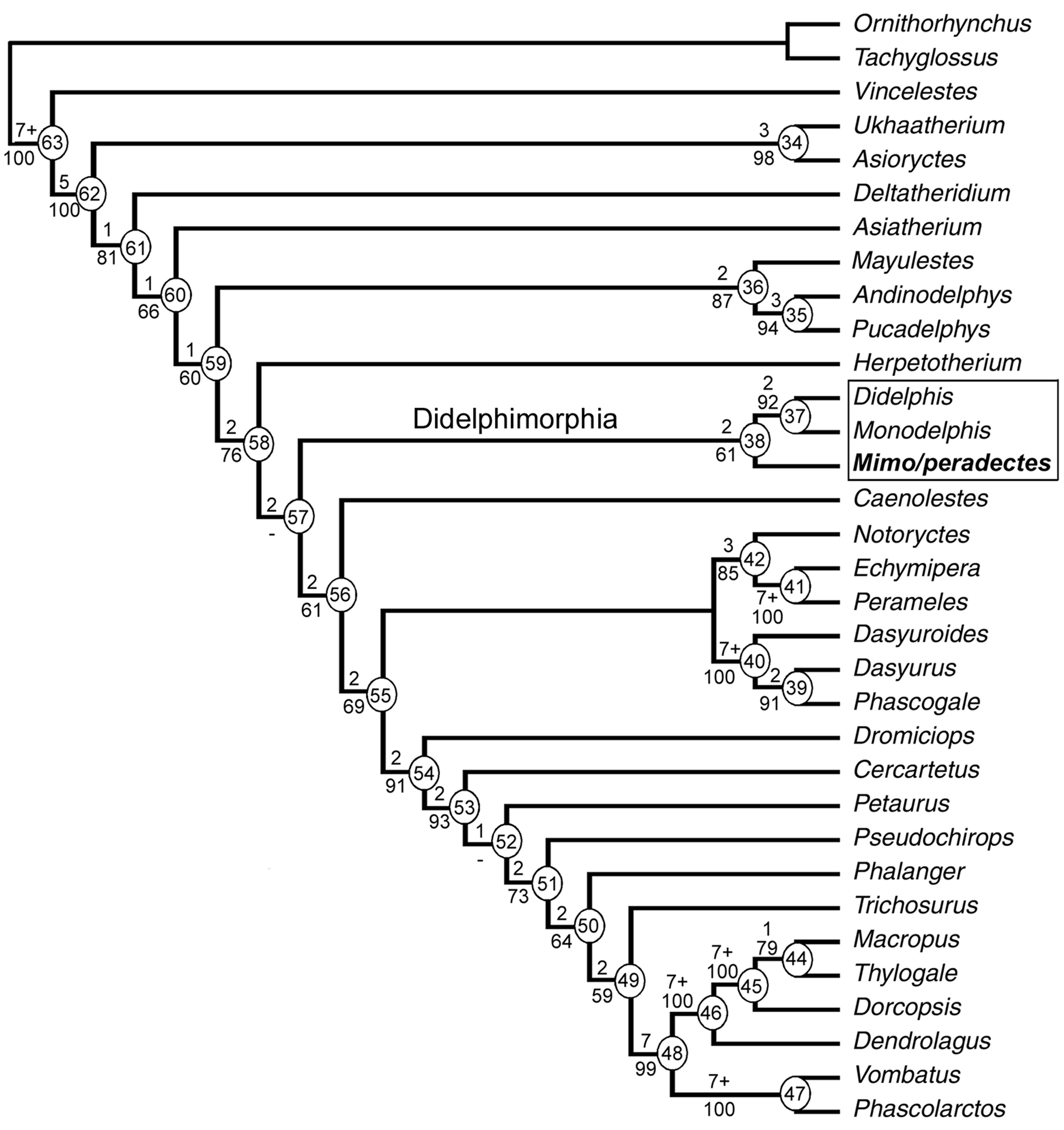

Supplement: Figure S6 — Most parsimonious cladogram resulting from analysis of morphological data matrix. Numbers inside circles identify nodes. Of the numbers outside circles, those that are above indicate values of Bremer support and those that are below indicate values of jackknife support with 25% deletion of characters. Branches that display values of jackknife support below 73, received no support in a bootstrap search with 50% deletion of characters. Note: Mimo/peradectes stands for Mimoperadectes-Peradectes (4.36 MB TIF) [file pone.0008278.s012.tif]
